# Inhibiting anti-angiogenic VEGF165b activates a miR-17-20a-Calcipressin-3 pathway that revascularizes ischemic muscle in peripheral artery disease

**DOI:** 10.1038/s43856-023-00431-5

**Published:** 2024-01-05

**Authors:** Sonia Batan, Sivaraman Kuppuswamy, Madison Wood, Meghana Reddy, Brian Annex, Vijay Ganta

**Affiliations:** 1 Vascular Biology Center, Department of Medicine, Augusta University, Augusta, GA 30912 USA; 2 Medical College of Georgia, Augusta University, Augusta, GA 30912 USA

**Keywords:** miRNAs, Angiogenesis, Peripheral vascular disease, Growth factor signalling, Blood flow

## Abstract

**Background:**

VEGF_165_a increases the expression of the microRNA-17-92 cluster, promoting developmental, retinal, and tumor angiogenesis. We have previously shown that VEGF_165_b, an alternatively spliced anti-angiogenic VEGF-A isoform, inhibits the VEGFR-STAT3 pathway in ischemic endothelial cells (ECs) to decrease their angiogenic capacity. In ischemic macrophages (Møs), VEGF_165_b inhibits VEGFR1 to induce S100A8/A9 expression, which drives M1-like polarization. Our current study aims to determine whether VEGF_165_b inhibition promotes perfusion recovery by regulating the microRNA(miR)-17-92 cluster in preclinical PAD.

**Methods:**

Femoral artery ligation and resection was used as a preclinical PAD model. Hypoxia serum starvation (HSS) was used as an in vitro PAD model. VEGF_165_b was inhibited/neutralized by an isoform-specific VEGF_165_b antibody.

**Results:**

Here, we show that VEGF_165_b-inhibition induces the expression of miR-17-20a (within miR-17-92 (miR-17-18a-19a-19b-20a-92) cluster) in HSS-ECs and HSS-Møs vs. respective normal and/or isotype-matched IgG controls to enhance perfusion recovery. Consistent with the bioinformatics analysis that revealed RCAN3 as a common target of miR-17 and miR-20a, Argonaute-2 pull-down assays showed decreased miR-17-20a expression and higher RCAN3 expression in the RNA-induced silencing complex of HSS-ECs and HSS-Møs vs. respective controls. Inhibiting miR-17-20a induced RCAN3 levels to decrease ischemic angiogenesis and promoted M1-like polarization to impair perfusion recovery. Finally, using STAT3 inhibitors, S100A8/A9 silencers, and VEGFR1-deficient ECs and Møs, we show that VEGF_165_b-inhibition activates the miR-17-20a-RCAN3 pathway independent of VEGFR1-STAT3 or VEGFR1-S100A8/A9 in ischemic-ECs and ischemic-Møs respectively.

**Conclusions:**

Our data revealed a hereunto unrecognized therapeutic ‘miR-17-20a-RCAN3’ pathway in the ischemic vasculature that is VEGFR1-STAT3/S100A8/A9 independent and is activated only upon VEGF_165_b-inhibition in PAD.

## Plain language summary

Therapies that can grow new blood vessels in the ischemic muscle are necessary to restore blood flow and provide relief to patients with peripheral artery disease (PAD). We have previously shown that blocking VEGF_165_b, a small protein involved in the regulation of regenerating blood vessels, promotes the growth of new blood vessels in the ischemic muscle. However, the mechanism by which this occurs is not clear. Here, we build on this existing knowledge and show the complex processes driving the growth of new blood vessels, which will help to supply blood to the ischemic muscle and provide therapeutic relief from PAD.

## Abstract (web summary)

Batan et al. show that inhibiting the antiangiogenic VEGF_165_b isoform activates a previously unrecognized miR-17-20a-RCAN3 pathway that induces ischemic endothelial cell angiogenic capacity and promotes M2-like macrophage polarization to achieve perfusion recovery in murine peripheral artery disease (PAD) model.

## Introduction

Peripheral artery disease (PAD) occurs due to atherosclerotic occlusions in the inflow blood vessels, resulting in decreased blood flow to the lower extremities (usually legs)^1^. In its most severe form, decreased skeletal muscle perfusion results in chronic limb-threatening ischemia (CLTI), which often results in impaired wound healing and necrosis leading to limb amputation^2^. Approximately 200,000 amputations/year occur in the US, with PAD being the major cause^3^. Approximately 20% of patients with CLTI are also at high risk for cardiovascular death within the first year of diagnosis^4,5^. Currently, no approved medical therapies can revascularize the ischemic muscle and promote perfusion relief in PAD patients, indicating a greater need to identify therapies that can promote limb perfusion in PAD patients.

Vascular endothelial growth factor-A (VEGF-A) is a well-known inducer of angiogenesis^6^. Clinical trials aimed at achieving perfusion recovery in patients with PAD by activating VEGF-A-VEGFR2 signaling in PAD were not successful^2^. An inadequate understanding of VEGF-A signaling in ischemic muscle vasculature could account for those failures. The signaling networks regulated by the VEGF-A family became more complex with the recognition of alternative splicing in the 8^th^ exon of VEGF-A that results in the formation of pro-angiogenic VEGFxxxa (xxx for the no. of amino acids. VEGF_165_a) and anti-angiogenic VEGFxxxb (VEGF_165_b) isoform families^7,8^. The only difference between these 2 isoforms is a 6-aminoacid switch from CDKPRR (in humans and mice) in the VEGF_165_a isoform to SLTRKD (PLTGKD in mice) in the VEGF_165_b isoform.

In our recent studies, we have shown that the fraction of anti-angiogenic VEGF_165_b isoforms is ~3-fold higher than the pro-angiogenic VEGF_165_a isoforms in human PAD muscle biopsies compared to age- and sex-matched controls. In preclinical PAD models, ischemia-induced ~6-fold higher numbers of VEGF_165_b^+^ ECs^9^ and ~15-fold higher VEGF_165_b^+^ macrophages^10^ (Møs) compared to nonischemic muscle. Using a highly isoform-specific monoclonal antibody raised against the 6-amino acids that neutralize the VEGF_165_b isoform (without affecting other pro-angiogenic VEGF_165_a isoforms^7–12^), we have shown that VEGF_165_b inhibition activates signal transducer and activator of transcription 3 (STAT3^13,14^); in ischemic ECs to increase their angiogenic capacity^9^ but inhibits S100 calcium-binding protein A8/A9 (S1008/S1009) expression in ischemic Møs (independent of STAT3 activation) to induce a reparative M2-like phenotype^9,10^. S100A8 and S100A9 (belonging to the S100 family of alarmins) are proinflammatory calcium-binding proteins that are highly expressed in neutrophils and macrophages^15^. Recent studies have shown that S100A8/A9 induces cell death in infarcted myocardium^16^ and inhibiting S100A9 improves cardiac function in myocardial infarction^17^. We have recently shown that VEGF_165_b inhibition decreases S100A8/A9 expression to induce an M2-like phenotype that promotes perfusion recovery^10^. Consistent with this data, Slayer et al.^18^, have shown that exogenous delivery of S100A8/A9 inhibited perfusion recovery in experimental PAD. Furthermore, we have shown that VEGF_165_b inhibition in ischemic muscle produces therapeutic perfusion recovery in multiple preclinical PAD models^9,10,12^, including endothelial nitric oxide synthase (eNOS)-KO, Myoglobin Transgenic, and Type-2 Diabetic PAD mice^12^. However, significant gaps remain in our understanding of the genetic regulators downstream of VEGF_165_b inhibition that regulate the extent of perfusion recovery in ischemic muscle, including whether the effects of the VEGF_165_b inhibition are STAT3 and/or S100A8/A9 dependent.

Noncoding RNAs are becoming attractive targets to treat human disease due to their ability to modulate the expression of multiple genes and biological pathways^19^. While lncRNAs are >200 base pairs in length and poorly conserved across species^20^, miRNAs are single-stranded ~16-23 nucleotide lengths that are relatively well conserved across species^21^. Despite recent advances in noncoding RNA biology, our understanding of the regulation or function of lncRNAs or miRNAs in human pathologies is still limited. In general, the pairing of the miRNA seed sequence to the 3’ UTR of the target gene within a ribonucleotide binding region promotes target gene degradation or translational inhibition^22,23^. miRNAs can occur as individuals or as a part of a miRNA cluster. For example, the miRNA-17-92 cluster (consisting of miR-17, -18, -19a, -19b, -20a, and -92a (92) members), also known as ‘OncomiR-1’, is one of the best-studied miRNA clusters due to its role in cancer biology^24,25^. While extensive literature is available on the miR-17-92 cluster in promoting tumor progression^25,26^, miR-92a and miR-19 within this cluster have been shown to inhibit perfusion recovery in PAD^27,28^. Hence, we wanted to determine the role of the VEGF_165_b-miR-17-92 cluster in regulating perfusion recovery in experimental PAD.

Here, our data shows that VEGF_165_b inhibition induces the expression of miR-17-20a in ischemic endothelial cells and in ischemic macrophages independent of VEGFR1-STAT3 or VEGFR1-S100A8/A9 signaling pathways. miR-17-20a targets and inhibits Calcipressin-3 (RCAN3, that inhibits tumor progression^29^ and HUVEC proliferation^30^) to increase ischemic endothelial angiogenic capacity and promote M2-like phenotype in ischemic macrophages. Activation of miR-17-20a-RCAN3 pathway increases endothelial cell proliferation and microvascular remodeling in ischemic skeletal muscle vasculature and enhances the perfusion recovery in experimental PAD.

## Methods

### Cell culture

Human umbilical vein endothelial cells (HUVECs) were purchased and cultured in an All-in-one complete endothelial growth medium (Cat No: 211-500) from Cell Applications Inc. San Diego, CA. HEK293 cells were cultured in DMEM supplemented with 10% FBS.

### Murine skeletal muscle microvascular endothelial cell isolation

Gastrocnemius and tibialis anterior muscle from individual male mouse hind limbs were collected, finely minced, and digested in endothelial basal medium supplemented with 1 mg/ml type-2 collagenase and 50 U/ml DNase for 40 min in a 37 °C orbital shaker at 250 rpm. The cell suspension was passed through 70 µM filters and centrifuged at 500 × *g* for 5 min. The cell pellet was washed once with PBS and incubated with mouse CD31-conjugated Dyna Beads for 1 h at 4 °C with gentle rocking. Later, magnetically bound SKMVECs were separated using a magnetic stand, washed, and suspended in mouse endothelial growth medium in 25 mm culture plates^9,10,31^. At 10-14 days of confluence, SkMVECs were trypsinized, and the CD31 isolation process was repeated to enrich for pure EC fractions. SkMVECs at this step were considered passage 2. ECs at passage 2 were immunoblotted for EC markers, including VEGFR2, VE-Cadherin, and PECAM (full-length western blots presented in Supplementary Fig. 1), and their ability to form tube-like structures on Matrigel. SkMVECs were used by passage 4.

### Murine bone marrow isolation and bone marrow-derived macrophage culture

Hind legs of individual male mice were flushed with phosphate-buffered saline, and the bone marrow cells were passed through a 70 µM filter and plated in culture in 10% DMEM supplemented with 10% L292 conditioned medium. The medium was changed the next day to remove nonadherent cells. Adherent cells were cultured in macrophage growth medium for 7-10 days with media changes every 2 days^10,31^.

### Hypoxia serum starvation

Macrophages or ECs were incubated in starvation buffer and subjected to hypoxia (2%, 6 h for macrophages; 24 h for ECs) according to the experimental conditions^9,10,31,32^.

### VEGF_165_b-Ab treatment

Cells were treated with 10 µg/ml isotype-matched IgG or VEGF_165_b-Ab for 24 h under HSS conditions.

### miR-17 and miR-20a overexpression in vitro

Cells were transfected with miRNA mimics (HUVECs-30 nM, BMDMs-150 nM) using siPORT^TM^ NeoFX^TM^ transfection reagent (Thermo Fisher Scientific, AM4511) according to the manufacturer’s protocol. Nontargeting negative mimics were used at the same concentration to account for off-target effects. Twenty-four hours post-transfection, cells were used in the experiments or challenged with HSS^31,32^.

### miR-17 and miR-20a inhibition in vitro

Cells were transfected with miRNA antagomirs (HUVECs-30 nM, BMDMs-150 nM) using siPORT^TM^ NeoFX^TM^ transfection reagent (Thermo Fisher Scientific, AM4511) according to the manufacturer’s protocol. Nontargeting negative antagomirs were used at the same concentration to account for off-target effects. 24 h post-transfection, and cells were used in the experiments or challenged with HSS^31,32^.

### Gene silencing in BMDMs

BMDMs were electroporated with equimolar nontargeting controls siRNAs or siRNAs against S100A8 or S100A9 using a Thermofisher Neon-electroporation unit according to the manufacturer’s protocol.

### STAT3 inhibition in ECs

HUVECs and SkMVECs were treated with S3I-201^33^ at 100uM or DMSO (equal volume) under normal or HSS conditions for 24 h followed by use in experiments.

### In vitro plasmid transfection

Cells were transfected with 1–2 µg of plasmid using Lipofectamine-3000 according to the manufacturer’s instructions^9,10,31^

### In vitro angiogenesis

Endothelial cells treated according to the experimental requirements and post-treatment were trypsinized, and an equal number of cells were plated on growth factor-reduced Matrigel (Thomas Scientific, Cat No: 356231, normal condition) in 96-well plates. Three to four hours after plating on the Matrigel, cells were observed under a bright field microscope, and images were obtained at the concave center of the Matrigel in 96-well plates. Capillary-like tubes formed on the Matrigel were counted manually by an observer blinded to the treatment conditions. Endothelial branching was measured by the Angiogenesis Analyzer in NIH ImageJ 1.48 v.

### 3’ UTR luciferase assay

HEK293 cells were transfected with 1–2 µg of RCAN3 3’-UTR luciferase-expressing plasmid (Cat No: SC208609, Origene) using Lipofectamine-3000. Seventy-two hours later, the cells were trypsinized and transfected with 150 nM negative mimic, miR-17 mimic, or miR-20a mimic using siPORT^TM^ NeoFX^TM^ transfection reagent. Twenty-four hours post-transfection, the cells were lysed, and a luciferase assay was performed using a Promega Luciferase assay kit (Cat No: E1500)^31,32^.

### Argonaute-2 immunoprecipitation

Normal or HSS-challenged HUVECs or BMDMs were lysed in RIPA lysis buffer supplemented with RNAse inhibitor (Murine, Cat No: M0314S (for BMDMs), Human, Cat No: M0307S (for HUVECs)) at a concentration of 50 U/ml. Cell lysates were precleared with 10 µl/sample Pierce™ Protein A/G Magnetic Beads (Cat No: 88802, ThermoFisher) for 1 h at 4 °C with end-end rotation. Precleared cell lysates were incubated with 5 µg of Argonaute-2 antibody (Cat No: ab186733, Abcam) for 4 h at 4 °C followed by incubation with 100 µl of goat-anti-rabbit magnetic beads (Cat No: 21356, ThermoFisher) for 1 h 30 min at 4 °C. After incubation, antigen-RNA bound magnetic beads were washed 3 times with autoclaved PBS (to remove RNases and DNases) supplemented with 25 U/ml of murine or human RNAase inhibitor using DynaMag™-2 Magnet (Cat No: 12321D, ThermoFisher). After the last wash, magnetic beads were resuspended in 200 µl of PBS supplemented with 25 U/ml RNase inhibitor. Then, 6x Reducing Laemli SDS-Sample Buffer (Cat No: BP-111R, Boston Bioproducts) was added to 50 µl of magnetic bead suspension and incubated at 95 °C for 5–10 min in an Eppendorf ThermoMixer set at 1000 rpm. Later, the magnetic beads were discarded using the Dynamag magnet, and the protein eluted into the sample buffer was used for western blot analysis of Argonaute-2^9,10^. Then, 500 µl of TRIzol was directly added to the remaining 150 µl of magnetic bead suspension and vortexed for 15-30 seconds at room temperature. The magnetic beads were discarded using a DynaMag magnet. The TRIzol suspension was used to isolate total RNA. Total RNA was used for mRNA and miRNA qPCR analysis.

### Mice

All animal experiments were approved by Augusta University’s Institutional Animal Care and Use Committee (IACUC) in adherence with National Institutes of Health guidelines on Humane Care and Use of Laboratory Animals. Animal experiments were performed under the IACUC-approved animal protocol number 2019-1004 but were exempted from obtaining Ethical approval from Augusta University’s Internal Review Board.

A total of 52 male mice were used for experiments that included laser scanning blood flow recovery, VEGF_165_b antibody administration, primarily skeletal muscle microvascular endothelial cell isolation, and developing bone marrow-derived macrophages. Healthy male 12–16-week-old C57BL/6J mice were used in all the experiments. Mice were housed in pathogen-free husbandry with free access to water and chow (Tekland, Cat No: 8064) with a 12 h light/dark cycle. Mice purchased from Jax were allowed to acclimatize for a minimum of a week before use in the experiments. Mice planned for this study were not used in any previous experiments. VEGFR1^+/-^ mice were developed by replacing the VEGFR1 signal sequence encoding the signal peptide with a lacZ-NEO cassette. While VEGFR1^+/-^ heterozygous mice are viable, VEGFR1^-/-^ results in embryonic lethality at E8.5. These mice have been used in our previous publications^9,10^ and have been donated to the Jackson laboratory (Strain: 022541, RRID: IMSR_JAX:022541). Mice from the same cage were randomly allocated as control or experimental groups by personnel performing the hind limb ischemia surgery. Each cage has mice that received control or experimental treatments at relatively equal numbers to minimize potential confounders such as the order of treatments/measurements, or animal/cage location. The surgeon performing the hind limb ischemia surgery and perfusion recovery measurements and necrosis incidence was aware of the group allocation throughout the study.

### Intramuscular plasmid delivery by electroporation

For RCAN3-expressing plasmid transfer into mouse hind limbs, electric-pulse-mediated gene transfer was performed. Briefly, under isoflurane anesthesia, 150 µg of RCAN3-expressing plasmid (150 µg/100 µl of saline) was injected into mouse hind limbs using a 0.5-ml syringe with a 28-gauge needle in 3 sites (1 site in TA and 2 nonoverlapping sites in GA). Seven electric pulses (100 ms, 1 Hz, and 100 V) were delivered immediately to the injected muscle using a 2-needle array (BTX Gemini Electroporator) placed on the medial and lateral sides of the muscle so that the electrical field was perpendicular to the long axis of the myofibers. Mice were allowed to recover for 7 days before use in HLI experiments^9,34^.

### Animal model of hind limb ischemia (HLI) and perfusion recovery

Mice were anesthetized with a combination of ketamine 90 mg/kg and xylazine 10 mg/kg. Unilateral femoral artery ligation and excision were performed on 12- to 16-week-old male mice (number as indicated for each experimental result) to induce hind limb ischemia, a widely used preclinical PAD model by several labs across the globe that closely recapitulates several pathological features of human PAD. Briefly, the femoral artery was ligated and resected from just above the inguinal ligament to its bifurcation at the origin of the saphenous and popliteal arteries. The inferior epigastric, lateral circumflex, and superficial epigastric artery branches were also ligated. Meloxicam (5 mg/kg) was injected once daily at 24 h intervals for 2 days to relieve pain and discomfort in the mice that underwent HLI surgery. The sex and age of the mice were matched in all experiments. Perfusion recovery was measured by quantifying microvascular blood flow by laser Doppler imaging (Perimed, Inc., Ardmore, PA) on days 0, 3, 7, and 14 post-HLI by personnel performing the HLI surgery. Perfusion in the ischemic limb was normalized to that in the nonischemic limb for each mouse^9,10,31,32,34^. Exclusion criteria set a priori included mice that showed more than 50% perfusion recovery by day 3 post-HLI. Adverse events included sudden death, lethargy, and severe weight loss.

### Necrosis scores

The extent of necrosis was scored as follows: Grade I: involving only toes, Grade II: extending to dorsum pedis, Grade III: extending to crus, and Grade IV: extending to the thigh or complete necrosis^9,10,31,32,34^. Exclusion criteria set a priori included mice that showed Grade IV necrosis by day 1 post-HLI. Mice with necrosis severity reaching Grade IV were sacrificed at the first sight of notice according to the animal care and use guidelines.

### Immunofluorescence

Animals were sacrificed, and the gastrocnemius muscle was immediately separated and fixed using 4% PFA at 4 °C for 48 h. Later, the tissues were incubated in 30% sucrose for an additional 24–48 h (until the tissues sank to the bottom), embedded in OCT compound, and cryopreserved. Cryosections (5 µM thick) were obtained from OCT-embedded tissues and immunostained by incubating in CD31 primary antibodies overnight at 4 °C followed by washes in PBS+0.05% Tween20 and incubating in secondary antibodies conjugated to Alexa Fluor-488 (Thermo Fisher Scientific, Cat No: A11006) or Alexa Fluor-555 (Thermo Fisher Scientific, A31572) for 2 h at room temperature. Sections were washed in PBS+0.05% Tween-20 and mounted using ProLong gold antifade mounting media (Thermo Fisher Scientific, P36935). The CD31 (Thermo Fisher, Cat No: ENMA3105)^35–37^, α-smooth muscle actin (Santa Cruz Biotechnology, Cat No: sc-130617)^38–40^, and PCNA (Millipore-Sigma, Cat No: NA03)^41–43^ antibodies used in this study have been extensively used and validated by our laboratory and others for their specific recognition of endothelial cells^35–37^, vascular smooth muscle cells^38–40^ and nuclear localization^41–43^, respectively, in tissue sections. Hence, tissue sections blocked with serum from the host of secondary antibodies followed by the omission of primary or secondary antibodies served as negative controls in our experiments. Immunofluorescence was visualized, and images were obtained using an EVOS M5000 fluorescence microscope^9,10,31^. Tissue collection, immunostainings, and data analysis were performed by personnel blinded to the experimental groups or perfusion recovery outcomes.

### Capillary density

Vascular density was quantified as %CD31+ cells per muscle fiber area (mm2) on day 14 or day 21 post-HLI in gastrocnemius muscle sections. At least 3 random images per section from each muscle tissue immunostained with CD31 and SMA were photographed. In each image, CD31^+^ cells/muscle fiber area were counted. Averages of CD31+ cells/muscle fiber area/tissue from different groups were plotted on GraphPad Prism9 and examined for statistical significance. Differences in fold change were obtained by normalizing the mean of the control group with the experimental group^9,10,31^.

### Endothelial proliferation

EC proliferation was quantified as % total CD31^+^ PCNA^+^ cell area (mm2) on day 14 or day 21 post-HLI sections. At least 3 random images per section from each muscle tissue immunostained with CD31 and PCNA antibodies were photographed. In each image, CD31^+^ PCNA^+^ cells were counted. Averages of CD31^+^ PCNA^+^ cells from different groups were plotted on GraphPad Prism9 and examined for statistical significance. Differences in fold change were obtained by normalizing the mean of the control group with the experimental group^9,10,31^.

### Western blotting

At least 50 μg of tissue or 10–20 μg of cell lysates were resolved by SDS‒PAGE and transferred onto nitrocellulose membranes for Western blotting^9^ using the iBright Imaging System.

### RNA isolation, cDNA preparation, and qPCR

Tissues or cells were lysed in TRIzol, and RNA was isolated using an Invitrogen™ PureLink RNA Mini Kit (Cat No: 12183025, ThermoFisher) according to the manufacturer’s instructions. RNA concentration was determined by NanoOne, and an equal amount of RNA was used to prepare cDNA using LunaScript® RT SuperMix (New England BioLabs, Cat no: M3010L) according to the manufacturer’s conditions. qPCR was performed by TaqMan primer probes using Luna Universal Probe qPCR Master Mix (New England Biolabs, Cat No: M3004) and Bio-Rad i-cycler according to standard lab procedures^9,10,31^. HPRT was used to normalize gene expression^31,32^. The ∆∆Ct method was used to calculate relative gene expression values in qPCR analysis.

### microRNA qPCR

Total RNA was isolated using Invitrogen™ PureLink™ RNA Mini Kit according to the manufacturer’s instructions followed by miR-17, -18a, 18-b, -19, -20a, -92a, and Sno202 specific reverse transcription to cDNA was performed using microRNA assay-specific primer probes using microRNA cDNA kit (Cat No: 4366596) and qPCR was performed using Taqman primer probes using Luna Universal Probe qPCR Master Mix (New England Biolabs, Cat No: M3004)^9,10,31^. SnoRNA202 was used to normalize the miR-17-92 cluster^31,32^.

### Statistics and reproducibility

GraphPad Prism9 was used to determine the statistical significance of the data and generate graphical representations. Data were analyzed for equal variance using the *F*-test. Data sets that passed the *F*-test were analyzed by unpaired *t*-test for 2-group comparisons or with one-way ANOVA followed by Bonferroni’s post-test for the comparison of multiple groups. Data sets that did not pass the *F*-test were analyzed by Unpaired *t*-test with Welch’s correction for 2 group comparisons or Brown-Forsythe one-way ANOVA with Welch’s correction for multiple group comparisons. Outliers were detected and removed by performing Grubb’s test. Perfusion recovery measurements were analyzed by two-way repeated-measures ANOVA with Bonferroni post-test. Necrosis scores were analyzed by the nonparametric Mann‒Whitney test. In vitro experiments were conducted with a minimum of *n* = 4 biological replicates (individual samples) and in vivo experiments were conducted with a minimum of *n* = 4 mice/group. All data are expressed as the mean  ±  standard error. *P* < 0.05 was considered significant. Statistical tests for each individual experiment are provided in the figure legend.

### Reporting summary

Further information on research design is available in the Nature Portfolio Reporting Summary linked to this article.

## Results

### Inhibiting the anti-angiogenic VEGF_165_b isoform induces truncated miR-17-20a expression (within the miR-17-92 cluster) in experimental PAD models

**Fig. 1:**
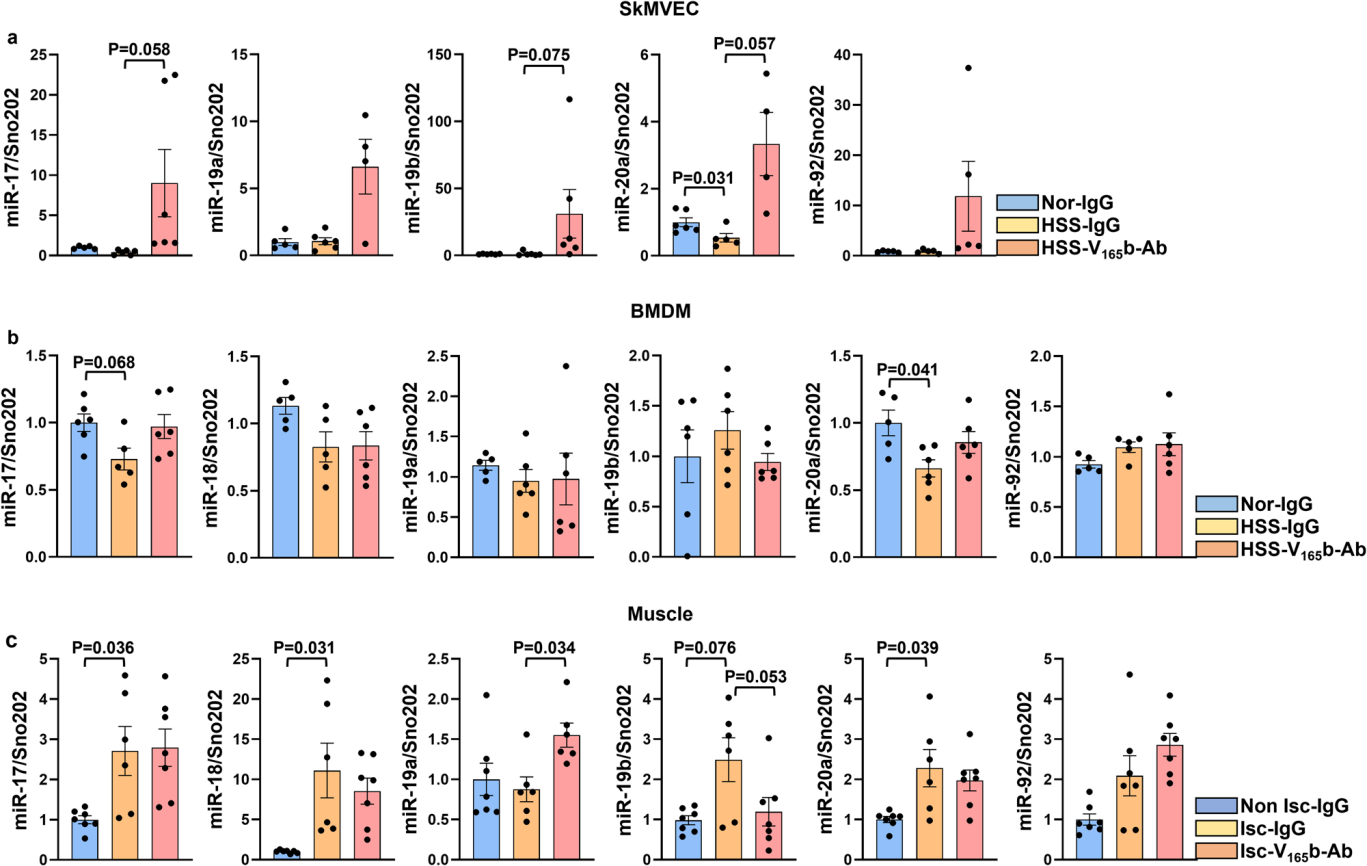
VEGF_165_b inhibition induces the expression of the miR-17-92 cluster in experimental PAD models. qPCR of miR-17-92 cluster (miR-17, miR-18, miR-19a, miR-19b, miR-20a, miR-92a) expression in **a** primary skeletal muscle microvascular ECs (SkMVECs, from Balb/CJ mice) under normal (Nor, blue bars) or hypoxic serum starvation (HSS) conditions for 24 h treated with IgG (yellow bars) or VEGF_165_b-Ab (pink bars). *n* = 6. One-way ANOVA with Bonferroni’s multiple comparisons for miR-17, miR-19a, miR-19b, and miR-92; Brown-Forsythe ANOVA with Welch’s correction for miR-20a. **b** Bone marrow-derived macrophages (BMDMs, from Balb/CJ mice) under Nor (blue bars) or HSS for 6 h treated with IgG (yellow bars) or VEGF_165_b-Ab (pink bars). *n* = 6. One-way ANOVA with Bonferroni’s multiple comparisons for miR-17, miR-18, miR-19a, miR-19b, miR-20a, and miR-92. **c** Nonischemic (Non Isc, blue bars) or ischemic (Isc) Balb/CJ mouse muscle treated with IgG (yellow bars) or VEGF_165_b-Ab (pink bars) at day 3 post-HLI. *n* = 7. Brown-Forsythe ANOVA with Welch’s correction for miR-17, miR-18, and miR-20a; One-way ANOVA with Bonferroni’s multiple comparisons for miR-19a, miR-19b, and miR-92. Outliers were removed by performing the Grubbs test. *P* < 0.05 significant. Data from the biological replicates are presented as mean ± standard error.

We wanted to determine whether VEGF_165_b inhibition modulates miR-17-92 cluster expression in ischemic ECs and/or Møs. qPCR analysis of skeletal muscle microvascular ECs (SkMVECs, isolated from pooled gastrocnemius and tibialis anterior muscle) showed that hypoxia serum starvation (HSS, an in vitro model for PAD^32^) significantly decreased miR-20a expression but did not affect the expression of other miR-17-92 cluster members compared to normal controls. However, VEGF_165_b inhibition (10 µg/ml) induced the expression of miR-17 (*P* = 0.058), miR-19b (*P* = 0.075) and miR-20a (*P* = 0.057) in HSS-SkMVECs compared to IgG (Fig. 1a). Interestingly, the expression of miR-18 in SkMVECs was very low (Ct values were higher than 35, suggesting very low to no expression, data not presented). To confirm whether the lack of miR-18 expression is confined to mouse primary SkMVECs, we used HUVECs that were previously used to determine the function of VEGF_165_b in HSS conditions in our publications^9^. qPCR analysis showed a significant decrease in the expression of miR-17, miR-18, miR-19a, miR-20a, and miR-92, but not miR-19b, in HSS-HUVECs vs. normal HUVECs (Supplementary Fig. 2), indicating a complete miR-17-92 cluster in human ECs. VEGF_165_b inhibition restored the expression of miR-17, miR-18, miR-19a, and miR-20a but not miR-92 to normal HUVEC levels in HSS-HUVECs vs. IgG (Supplementary Fig. 2). We next examined the role of VEGF_165_b inhibition in regulating miR-17-92 cluster expression in bone marrow-derived macrophages (BMDMs). qPCR analysis showed that HSS numerically decreased the expression of miR-17 (*P* = 0.068) and significantly decreased miR-20a expression, but not the expression of other cluster members compared to normal BMDMs. Inhibiting VEGF_165_b in HSS-BMDMs restored the expression of both miR-17 and miR-20a to normal BMDM levels (Fig. 1b).

To determine whether inhibiting VEGF_165_b induces the expression of miR-17-92 cluster members (miR-17, -18, -19a, -19b, -20a, and -92) in vivo, we performed qPCR analysis of Balb/cJ mice (an inbred mouse strain with poor perfusion recovery post-HLI) ischemic muscle treated with IgG or VEGF_165_b-Ab (200 µg/100 µl PBS, i.m, 2 nonoverlapping sites in GA and 1 site in TA) at day 3 post-HLI. qPCR analysis showed differential expression within the members of this cluster in ischemic muscle vs. nonischemic muscle. A significant increase in miR-17, miR-18, and miR-20a and a numerical increase in miR-19b (*P* = 0.076) expression were observed in ischemic vs. nonischemic muscle. No significant differences in miR-19a or miR-92 expression were observed in ischemic vs. nonischemic muscle. VEGF_165_b inhibition in ischemic muscle induced miR-19a expression without affecting the expression of other cluster members vs. IgG (Fig. 1c). Interestingly, contrary to Balb/cJ ischemic muscle treated with VEGF_165_b-Ab (Fig. 1c), VEGF_165_b inhibition in C57BL/6J mice (an inbred mouse strain with good perfusion recovery post-HLI) ischemic muscle induced the expression of miR-17 and miR-20a with no changes in miR-19a expression vs. ischemic muscle treated with IgG (Supplementary Fig. 3), suggesting that the strain of mice differentially regulates the expression of the miR-17-92 cluster to VEGF_165_b inhibition in ischemic muscle. No significant differences in other cluster members were observed between VEGF_165_b-Ab vs. IgG-treated C57BL/6 J ischemic-muscle samples (Supplementary Fig. 3). Based on these data, we hypothesized that VEGF_165_b inhibition induces the expression of the miR-17-20a cluster in ischemic ECs and Møs to promote perfusion recovery.

To determine whether STAT3 activation downstream of VEGF_165_b inhibition regulates miR-17-20a in ischemic ECs, we treated HUVECs and SkMVECs with a STAT3 inhibitor (S3I-201^33^) according to our previous publications^44,45^. STAT3 inhibition did not significantly induce any changes in miR-17-20a expression in either normal- or HSS-HUVECs vs. the respective controls (Supplementary 4a, b). However, while STAT3 inhibition induced miR-17-20a expression in normal SkMVECs (Supplement 4c), no significant difference in miR-17-20a expression was observed in HSS-SkMVECs (Supplement 4d). Since we have previously shown that inhibiting VEGF_165_b induces VEGFR1 activation, which activates STAT3 in HSS-ECs, we wanted to determine whether VEGFR1 regulates the miR-17-20a cluster independent of STAT3. However, no significant differences in miR-17-20a expression were observed in either normal or HSS-SkMVECs isolated from VEGFR1^+/-^ (VEGFR^-/-^ is embryonic lethal; and VEGFR1^+/-^ mice cannot upregulate VEGFR1 in ischemic muscle^9^, HSS-SkMVECs (Supplementary Fig. 5) or HSS-BMDMs^10^ compared to respective VEGFR1^+/+^ controls). vs. VEGFR1^+/+^ mice (Supplementary Fig. 6a, b), These data indicated that VEGF_165_b inhibition induces miR-17-20a cluster expression in HSS-ECs independent of VEGFR1-STAT3 signaling.

To determine whether S100A8/A9 plays a role in regulating miR-17-20a in ischemic Møs, we silenced S100A8 or S100A9 (Supplementary Fig. 7a, b) in normal or HSS-BMDMs and examined miR-17-20a expression. Silencing S100A8 or S100A9 did not affect miR-17-20a expression in normal or HSS-BMDMs (Supplementary Fig. 7c, d), indicating that VEGF_165_b inhibition induces miR-17-20a expression independent of S100A8/A9 in HSS-BMDMs. Next, we wanted to determine whether VEGFR1 regulates the miR-17-20a cluster independent of S100A8/A9^10^ in HSS-BMDMs. qPCR analysis of miR-17-20a expression showed no significant difference in miR-17-20a expression in normal or HSS-challenged VEGFR1^+/-^ vs. VEGFR1^+/+^ BMDMs (Supplementary Fig. 8a, b). These data indicated that inhibiting VEGF_165_b induces miR-17-20a expression in HSS-BMDMs independent of VEGFR1-S100A8/A9 signaling.

### The miR-17-20a cluster regulates perfusion recovery in PAD

**Fig. 2:**
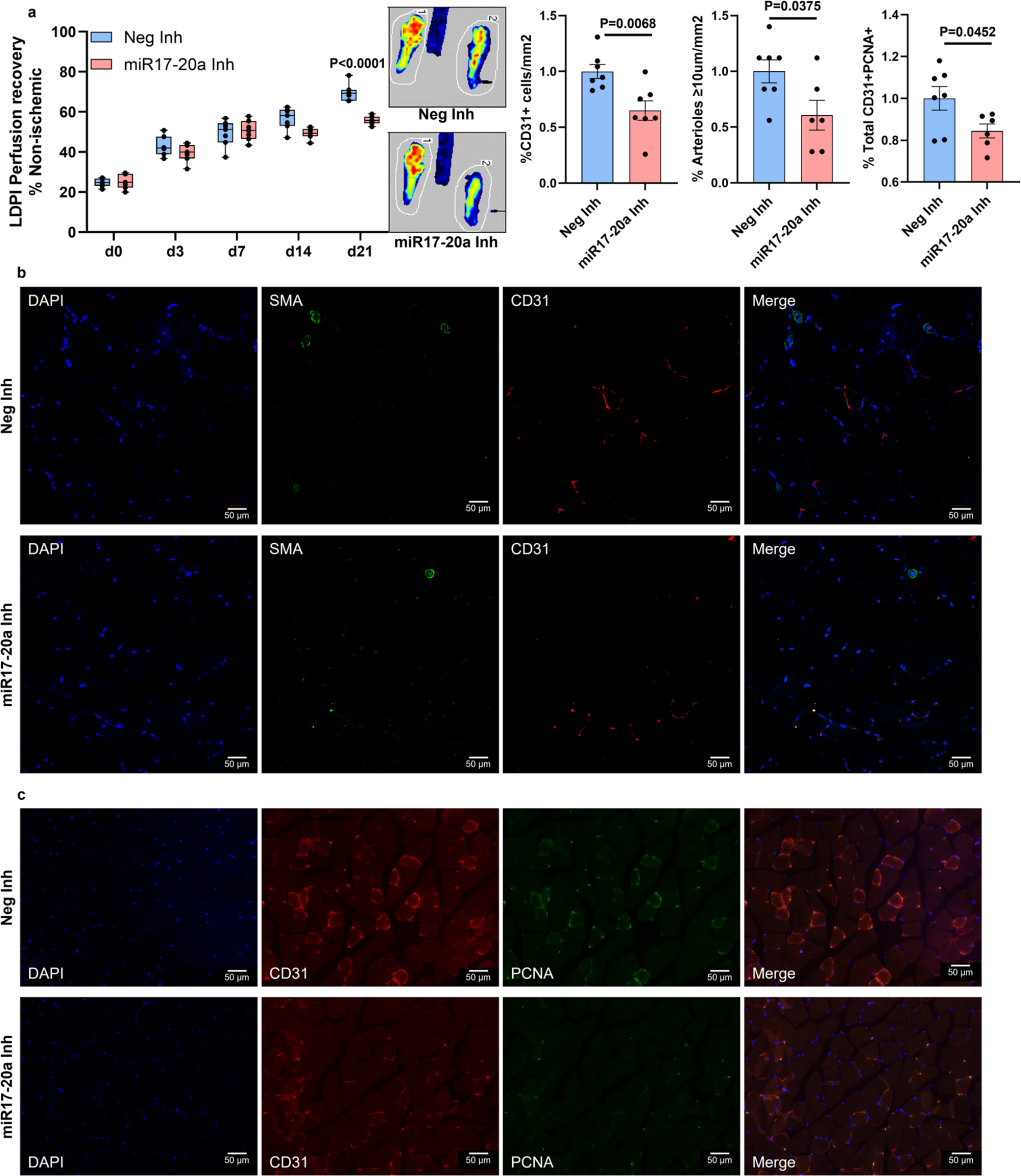
Inhibiting miR-17-20a decreases ischemic muscle revascularization in experimental PAD. **a** Laser Doppler perfusion imaging of microvascular blood flow in the ischemic muscle treated with a combination of miR-17, miR-20a inhibitor (miR-17-20a-Inh, pink box plots), or control inhibitor (Neg-Inh, blue box plot). *n* = 7. Repeated-measures Two-way ANOVA with Bonferroni’s post-test. **b** Immunohistochemical analysis of SMA (green) and CD31 (red) in ischemic gastrocnemius muscle treated with Neg-Inh (blue bars) or miR-17-20a-Inh (pink bars) at day 21 post-HLI. *n* = 7. Unpaired *t*-test. Scale bars are 50 µm. **c** Immunohistochemical analysis of CD31 (red) and PCNA (green) in ischemic gastrocnemius muscle treated with Neg-Inh (blue bars) or miR-17-20a-Inh (pink bars) at day 21 post-HLI. *n* = 7. Unpaired *t*-test. Scale bars are 50 µm. Outliers were removed by performing the Grubbs test. *P* < 0.05 significant. Data from the biological replicates are presented as mean ± standard error.

Since VEGF_165_b inhibition induced miR-17-20a expression in C57BL/6J ischemic muscle (Supplementary Fig. 3), we hypothesized that the ability of C57BL/6J ischemic muscle to induce miR-17-20a expression upon VEGF_165_b inhibition enhances perfusion recovery in experimental PAD. To test this hypothesis, we inhibited miR-17 and miR-20a in C57BL/6 J skeletal muscle by i.m. delivery of miR-17+miR-20a antagomirs (100 µM miR-17 and miR-20a antagomir or equimolar concentration of nontargeting antagomir (inhibitor) in 100 µl of PBS injected at 2 nonoverlapping sites in GA and 1 site in TA) at days 0, 3, 7, 14, and 21 post-HLI^9,10,31^ (Supplementary Fig. 9). miR-17-20a antagomirs significantly decreased perfusion recovery in C57BL/6J ischemic muscle vs. control inhibitor (day 21 post-HLI: control inhibitor-69.69 ± 1.9 vs. miR-17-20a inhibitor-56.04 ± 0.9, *P* < 0.0001, Fig. 2a). Immunohistochemistry of CD31 and α-smooth muscle actin (SMA, ≥10 µm vessels) showed a significant decrease (1.5-fold) in EC numbers and SMA^+^ arterioles in C57BL/6J ischemic muscle treated with miR-17-20a inhibitors vs. control inhibitors at day 21 post-HLI (Fig. 2b). Consistent with lower EC numbers, immunohistochemical analysis revealed a significant decrease in the fraction of proliferating ECs (PCNA^+^CD31^+^ in total PCNA^+^ cells) in miR-17-20a inhibitor-treated C57BL/6J ischemic muscle vs. control inhibitor at day 21 post-HLI (Fig. 2c). No significant difference in the necrosis incidence/scores was observed between C57BL/6J ischemic muscle treated with control inhibitor vs. miR-17-20a inhibitor.

**Fig. 3:**
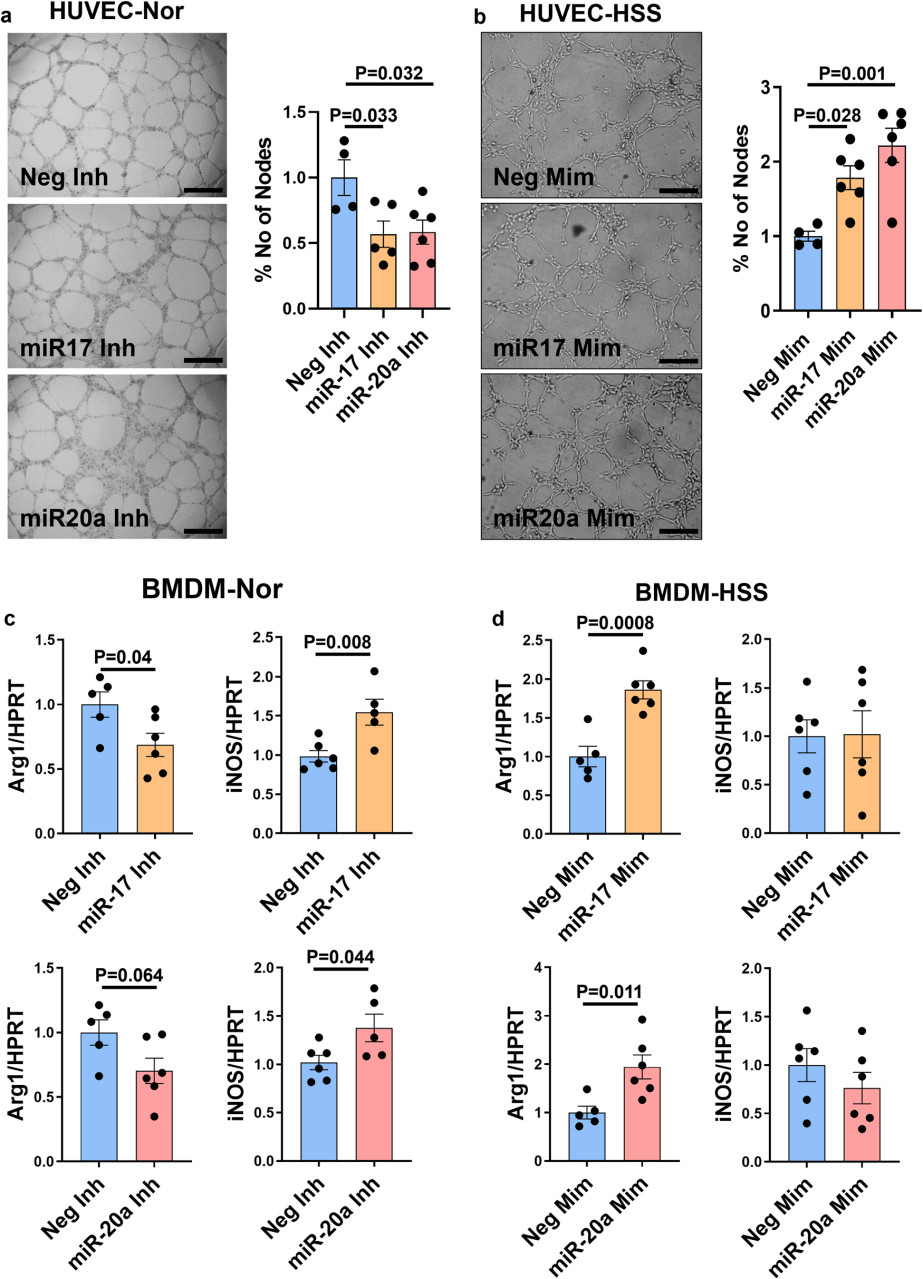
miR-17-20a induces ischemic angiogenesis and an M2-like macrophage phenotype. **a** In vitro tube formation assay of normal HUVECs transfected with negative inhibitor (Neg-Inh, blue bars, *n* = 4), miR-17 inhibitor (miR-17 Inh, yellow bars, *n* = 5) or miR-20a inhibitor (miR-20a Inh, pink bars, *n* = 6) on growth factor-reduced Matrigel (GFRM). Scale bars are 50 µm. One-way ANOVA with Dunnett’s post-test. **b** In vitro tube formation assay of HSS-HUVECs transfected with Neg-Mim (blue bars, *n* = 4), miR-17-Mim (yellow bars, *n* = 6), or miR-20a-Mim (pink bars, *n* = 6) on GFRM. *n* = 6. Scale bars are 50 µm. One-way ANOVA with Dunnett’s post-test. **c** qPCR of arginase-1 (Arg1) and inducible nitric oxide synthase (iNOS) expression in normal BMDMs transfected with negative inhibitor (Neg-Inh, blue bars), miR-17 inhibitor (miR-17 Inh, yellow bars,) or miR-20a inhibitor (miR-20a Inh, pink bars). *n* = 6. Unpaired *t-*test. **d** qPCR analysis of Arg1 and iNOS expression in HSS-BMDMs transfected with Neg-Mim (blue bars), miR-17-Mim (yellow bars), or miR-20a-Mim (pink bars). *n* = 6. Unpaired *t-*test. Outliers were removed by performing the Grubbs test. *P* < 0.05 significant. Data from the biological replicates are presented as mean ± standard error.

We next wanted to determine whether the increased angiogenic capacity in ischemic ECs post VEGF_165_b inhibition is mediated by increased miR-17-20 expression. We performed gain-of-function and loss-of-function experiments by inhibiting miR-17 and miR-20a in normal HUVECs (Supplementary Fig. 10a) and overexpressing miR-17 and miR-20a in HSS-HUVECs (Supplementary Fig. 10b). While normal HUVECs transfected with miR-17 or miR-20a antagomirs showed a significant decrease in tube-like formation on growth factor-reduced Matrigel (GFRM, control inhibitor: 129.5 ± 17.53, miR-17 inhibitor: 73.6 ± 12.9, miR-20a inhibitor: 75.67 ± 11.9, *P* < 0.05, Fig. 3a), HSS-HUVECs transfected with miR-17 or miR-20a mimics showed a significant increase in tube-like structures on GFRM (control mimic: 83.75 ± 5.7, miR-17-mimic: 149.5 ± 13.4, miR-20a mimic: 185.8 ± 19.2, *P* < 0.05, Fig. 3b) vs. the respective controls, indicating that the miR-17-20a cluster induces ischemic EC angiogenic capacity.

Since VEGF_165_b inhibition induces an M2-like phenotype in ischemic Møs, we next wanted to determine the role of miR-17-20a in regulating ischemic Mø polarization^10,31^. Gain-of-function and loss-of-function experiments were performed by inhibiting miR-17 and miR-20a in normal BMDMs (Supplementary Fig. 11a) and overexpressing miR-17 and miR-20a in HSS-BMDMs (Supplementary Fig. 11b). qPCR analysis showed no significant differences in Arg1 expression (M2 marker) but a significant increase in iNOS expression (M1 marker) in normal BMDMs transfected with miR-17 or miR-20a antagomirs (Fig. 3c). In HSS-BMDMs transfected with miR-17 or miR-20a mimics, a significant increase in Arg1 expression without any changes in iNOS expression was observed (Fig. 3d). These data indicated that the miR-17-20a cluster induces an M2-like-reparative phenotype in ischemic Møs.

### miR-17-20a targets RCAN3 in ischemic vasculature

**Fig. 4:**
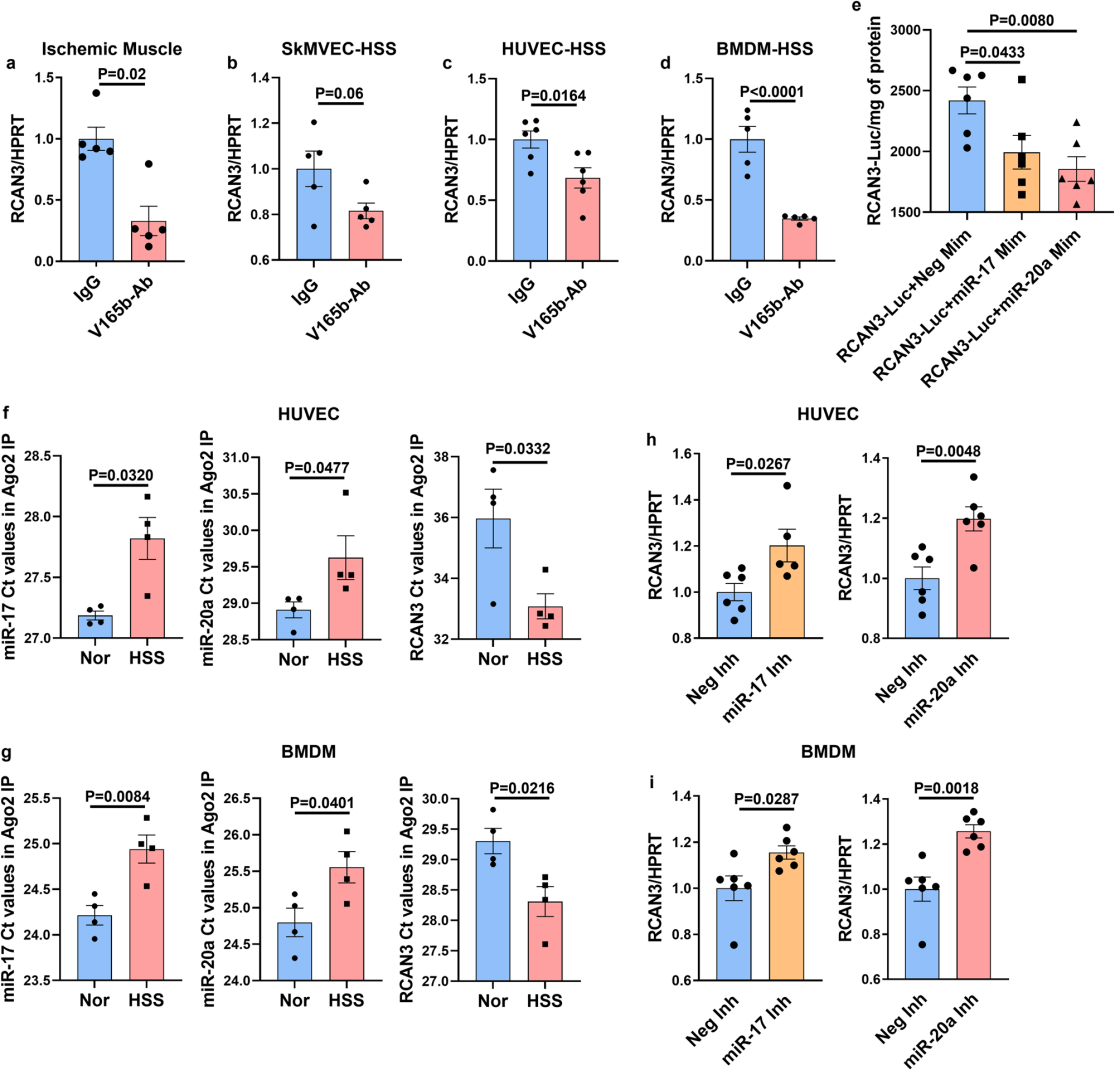
miR-17-20a targets RCAN3 in ischemic endothelial cells and macrophages. qPCR of RCAN3 expression in **a** IgG- (blue bars) or V_165_b-Ab-treated (pink bars) ischemic muscle. *n* = 5. Unpaired *t*-test. **b** IgG- (blue bars) or V_165_b-Ab-treated (pink bars) HSS-SkMVECs. *n* = 5. Unpaired *t*-test. **c** IgG- (blue bars) or V_165_b-Ab-treated (pink bars) HSS-HUVECs. *n* = 6. Unpaired *t*-test. **d** IgG- (blue bars) or V_165_b-Ab-treated (pink bars) HSS-BMDMs. *n* = 6. Unpaired *t*-test. *P* < 0.05 significant. **e** 3’ UTR luciferase assay in HEK293 cells transfected with RCAN3 3’UTR luciferase plasmid followed by transfection with Neg-Mim (blue bars), miR-17-Mim (yellow bars), or miR-20a-Mim (pink bars). *n* = 6. One-way ANOVA with Dunnett’s post-test. **f** qPCR analysis of miR-17 (unpaired *t*-test with Welch’s correction), miR-20a, and RCAN3 Ct values in Ago2-IP fractions from normal (blue bars) and HSS (pink bars) HUVECs. *n* = 4. Unpaired *t*-test. **g** qPCR analysis (Ct values) of miR-17, miR-20a, and RCAN3 expression in RCAN3-IP fractions from normal (blue bars) and HSS (pink bars) BMDMs. *n* = 4. Unpaired *t*-test. **h**, **i** qPCR of RCAN3-expression in **h** HSS-HUVECs and, **i** HSS-BMDMs transfected with negative inhibitor (Neg-Inh, blue bars), miR-17 inhibitor (miR-17-Inh, yellow bars) or miR-20a inhibitor (miR-20a Inh, pink bars). *n* = 6. Unpaired *t-*test. Outliers were removed by performing the Grubbs test. *P* < 0.05 significant. Data from the biological replicates are presented as mean  ±  standard error.

Since miRNA binding to their target genes results in transcriptional or translational inhibition^22^, we next wanted to determine the gene target of miR-17 and/or miR-20a that regulates perfusion recovery post-VEGF_165_b inhibition in ischemic muscle. Based on the bioinformatics analysis or miR-17 and miR-20a predicted targets using the mirdb target prediction database^46,47^ and Targetscan7.2^48^ that identified RCAN3 as a potential common target of miR-17 and miR-20a, we first examined whether VEGF_165_b inhibition modulates RCAN3 expression in experimental PAD models. qPCR analysis showed a significant decrease in RCAN3 expression in ischemic muscle (Fig. 4a), HSS-ECs (Fig. 4b), HSS-HUVECs (Fig. 4b) and HSS-BMDMs (Fig. 4d) treated with VEGF_165_b-Ab vs. respective IgG controls, indicating that VEGF_165_b inhibition decreases RCAN3 expression in experimental PAD models.

To obtain direct evidence of miR-17 and miR-20a targeting RCAN3, we performed a RCAN3-3’ UTR luciferase assay by transfecting HEK293 cells with RCAN3-3’UTR-Luc followed by transfection with miR-17 or miR-20a mimics. A luciferase assay showed a significant decrease in luciferase activity in miR-17- and miR-20a-transfected RCAN3-3’UTR-Luc HEK293 cells, indicating that miR-17 and miR-20a bind and inhibit RCAN3 expression (Fig. 4e). Furthermore, Argonaute-2 immunoprecipitated complexes in normal vs. HSS-HUVECs (Supplementary Fig. 12a) and normal vs. HSS-BMDMs (Supplementary Fig. 12b) showed a significant decrease in RCAN3 Ct values (indicating higher expression) and significantly higher miR-17 and miR-20a Ct values (indicating lower expression) in HSS-HUVECs (Fig. 4f) and HSS-BMDMs (Fig. 4g) vs. respective normal controls. Consistent with these findings, qPCR analysis of HSS-HUVECs transfected with miR-17 or miR-20a antagomirs showed a significant increase in RCAN3 expression vs. control antagomir (Fig. 4h) and a significant increase in RCAN3 expression in HSS-BMDMs transfected with miR-17 or miR-20a antagomir vs. control inhibitor (Fig. 4i). These data indicated that VEGF_165_b inhibition induces miR-17-20a expression in the ischemic vasculature, which inhibits RCAN3 expression to promote perfusion recovery in PAD.

### RCAN3 regulates ischemic endothelial angiogenic capacity and macrophage polarization

**Fig. 5:**
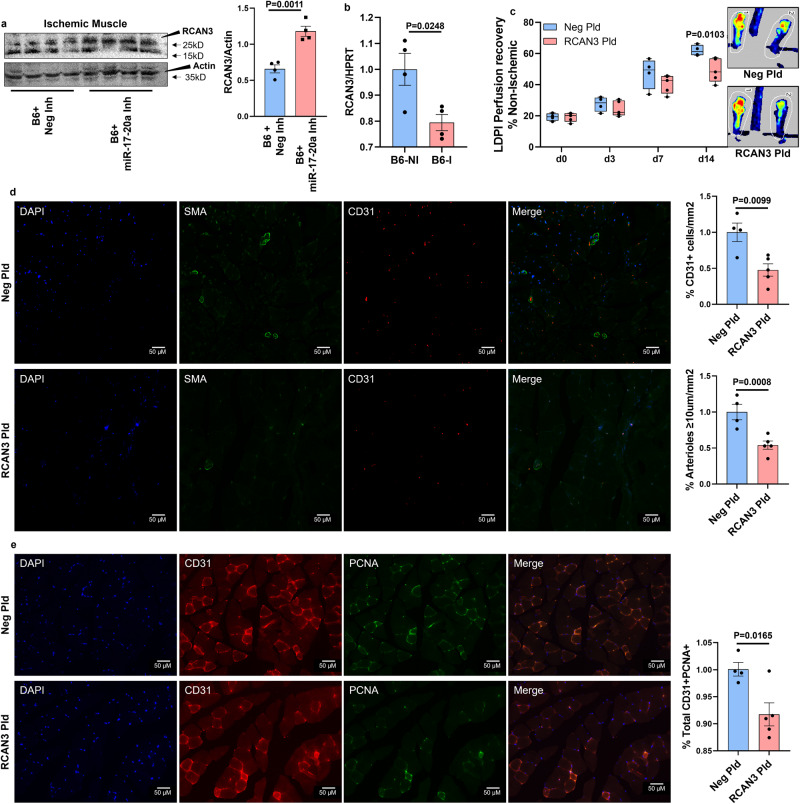
RCAN3 inhibits perfusion recovery in experimental PAD. **a** Western blot analysis of RCAN3 in ischemic muscle treated with negative inhibitor (Neg-Inh, blue bar) or a combination of miR-17 and miR-20a inhibitor (miR-17-20 Inh, pink bar). *n* = 4. Unpaired *t*-test. **b** qPCR of RCAN3 expression in C57BL/6J nonischemic (NI, blue bar) and ischemic (I, pink bar) muscle. *n* = 4. Unpaired *t*-test. **c** Laser Doppler perfusion imaging of microvascular blood flow in ischemic muscle transfected with a control plasmid (Neg Pld, blue box plots, *n* = 4) or RCAN3-expressing plasmid (RCAN3 Pld, pink box plots, *n* = 5). Repeated-measures two-way ANOVA with Bonferroni’s post-test. **d** Immunohistochemical analysis of SMA (green) and CD31 (red) in ischemic gastrocnemius muscle treated with Neg Pld (*n* = 4, blue bars) or RCAN3 Pld (*n* = 5, pink bars) at day 14 post-HLI. Scale bars are 50 µm. Unpaired *t*-test. **e** Immunohistochemical analysis of CD31 (red) and PCNA (green) in ischemic gastrocnemius muscle treated with Neg Pld (*n* = 4, blue bars) or RCAN3 Pld (*n* = 5, pink bars) at day 14 post-HLI. Scale bars are 50 µm. Unpaired *t*-test. *P* < 0.05 significant. Data from the biological replicates are presented as mean  ±  standard error.

Limited information exists on the role of RCAN3 in regulating angiogenesis in general or in PAD. First, we determined an in vivo role of miR-17-20a in regulating RCAN3 in preclinical PAD. Since C57BL/6J mice can upregulate miR-17-20a expression in ischemic muscle (Supplementary Fig. 3), we treated C57BL/6J mice ischemic muscle with control or miR-17-20a inhibitors (100 µM, i.m. 3 sites in muscle (2 in GA and 1 in TA) at day 0 and examined RCAN3 expression at day 3 post-HLI. Western blot analysis showed that miR-17-20a inhibition in C57BL/6 J ischemic muscle significantly induced RCAN3 levels compared to the negative inhibitor at day 3 post-HLI (Fig. 5a and Supplementary Fig. 13). Subsequent qPCR analysis showed a significant decrease in RCAN3 expression in C57BL/6J ischemic muscle vs. nonischemic muscle (Fig. 5b). Taken together, these data indicated that the ability of C57BL/6J mice to induce miR-17-20a expression in ischemic muscle targets RCAN3 to promote perfusion recovery.

To test the role of RCAN3 in regulating PAD, we induced RCAN3 expression by electroporating an RCAN3-expressing plasmid (vs. control plasmid, Supplementary Fig. 14) into C57BL/6J mice skeletal muscle (GA and TA) and performed HLI. Laser Doppler showed a significant decrease in perfusion recovery in C57BL/6 J mice ischemic muscle treated with RCAN3-expressing plasmid vs. control plasmid (day 14: control plasmid 61.9 ± 1.74 vs. RCAN3 plasmid 49.8 ± 3.4), indicating that increased RCAN3 levels impair perfusion recovery in PAD (Fig. 5c). Immunohistochemical analysis of CD31 and SMA (≥10 µm vessels) showed a significant decrease (~2-fold) in EC numbers and SMA+ arterioles in the ischemic muscle transfected with RCAN3-expressing plasmid vs. control at day 14 post-HLI (Fig. 5d). Consistent with the lower EC numbers, RCAN3 overexpression decreased the fraction of proliferating ECs (PCNA^+^CD31^+^ in total PCNA^+^ cells) vs. control plasmid at day 14 post-HLI (Fig. 5e). No significant difference in the necrosis incidence/scores was observed between C57BL/6J ischemic muscle treated with control plasmid vs. RCAN3-expressing plasmid.

**Fig. 6:**
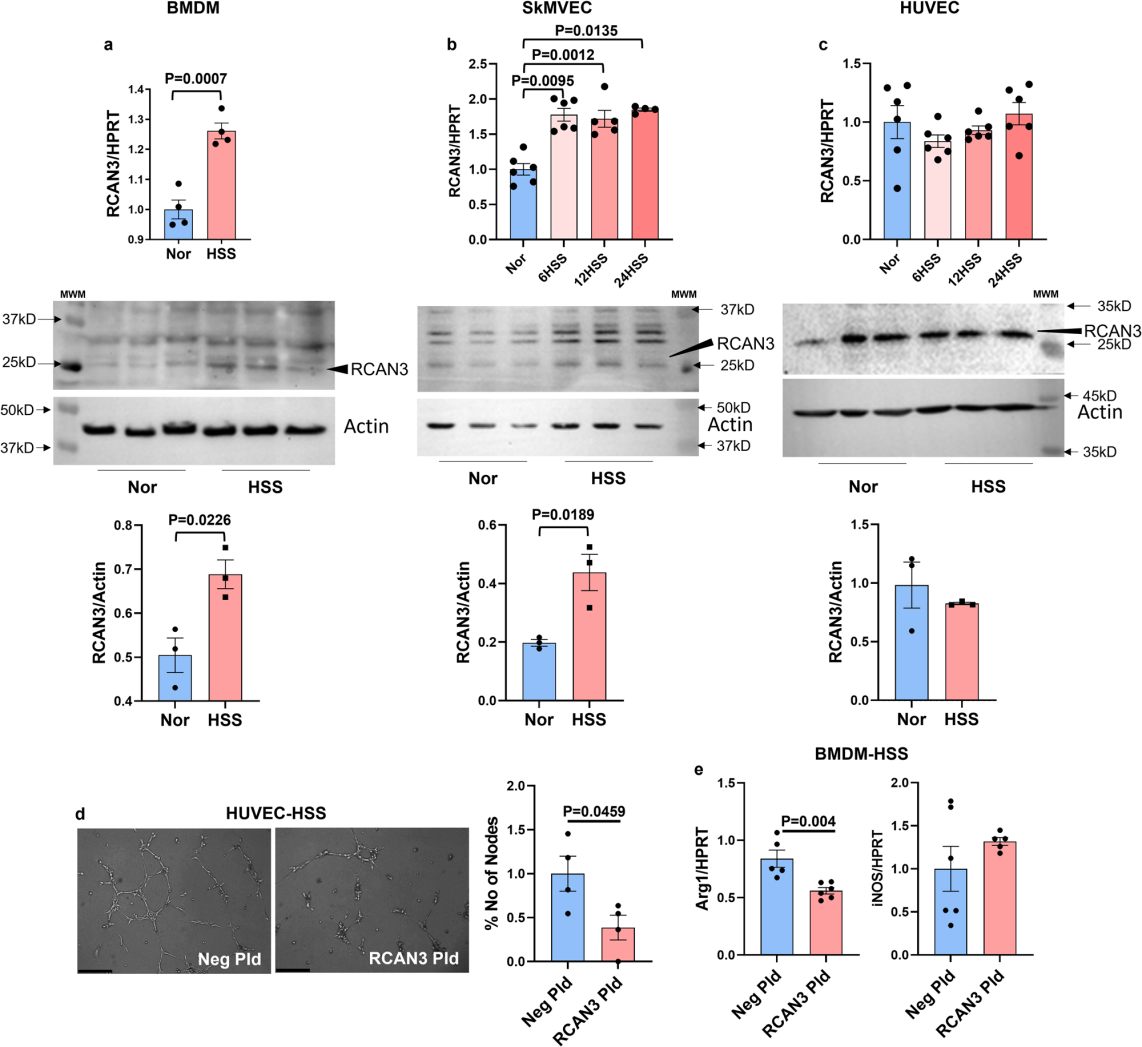
RCAN3 inhibits ischemic angiogenesis and induces an M1-like macrophage phenotype in vitro. qPCR analysis and western blot analysis of RCAN3 in **a** BMDMs (qPCR: normal *n* = 4 (blue bar), HSS *n* = 4 (pink bar); Western blot: normal *n* = 3 (blue bar), HSS *n* = 3 (pink bar), Unpaired *t*-test, **b** SkMVECs (qPCR: normal *n* = 6 (blue bar), HSS *n* = 6/time point (pink bars); Western blot: normal *n* = 3 (blue bar), HSS *n* = 3 (pink bar)), one-way ANOVA with Dunnett’s post-test for qPCR analysis, Unpaired *t*-test for western blot analysis, and **c** HUVECs (qPCR: normal *n* = 6 (blue bar), HSS *n* = 6/time point (pink bars), Western blot: normal *n* = 3 (blue bar), HSS *n* = 3(pink bar)), one-way ANOVA with Dunnett’s post-test for qPCR analysis, Unpaired-*t*-test for western blot analysis. **d** In vitro tube formation assay of HSS-HUVECs transfected with Neg Pld (blue bar) or RCAN3 plasmid (pink bar) on GFRM. *n* = 4. Scale bars are 50 µm. Unpaired *t*-test. **e** qPCR of arginase-1 (Arg1, (Unpaired *t*-test with Welch’s correction)) and inducible nitric oxide synthase (iNOS) expression in HSS-BMDMs transfected with Neg Pld (blue bar) or RCAN3 Pld (pink bar), *n* = 6. Unpaired *t*-test. *P* < 0.05 significant. Data from the biological replicates are presented as mean ± standard error.

We next wanted to determine the cell-specific function of RCAN3 in regulating ischemic-EC angiogenic capacity and ischemic-Mø polarization. qPCR and western blot analysis showed a significant increase in RCAN3 levels in HSS-BMDMs (Fig. 6a and Supplementary Fig. 15) and HSS-SkMVECs (Fig. 6b and Supplementary Fig. 15) vs. the respective normal controls. No significant difference was observed between normal vs. HSS-HUVECs (Fig. 6c and Supplementary Fig. 15). However, functionally, overexpressing RCAN3 (Supplementary Fig. 16a) significantly decreased HSS-EC tube-like formation on GFRM vs. control plasmid (Fig. 6d). RCAN3 overexpression (Supplement 16b) significantly decreased Arg1 expression without changing iNOS expression^10,31^ in HSS-BMDMs, indicating induction of the M1-like phenotype (Fig. 6e). These data indicated that RCAN3 inhibits ischemic EC angiogenic capacity and induces an M1-like cytotoxic phenotype to impair perfusion recovery in PAD.

Finally, we wanted to determine whether VEGFR1-STAT3 or VEGFR1-S100A8/A9 signaling regulates RCAN3 expression in ischemic ECs or ischemic Møs. STAT3 inhibition did not induce any significant changes in RCAN3 expression in normal or HSS-HUVECs (Supplement 17a) or in normal or HSS-SkMVECs (Supplement 17b). No significant differences in RCAN3 expression were observed in HSS-SkMVECs from VEGFR1^+/-^ vs. VEGFR1^+/+^ mice (Supplement 17c). Furthermore, silencing S100A8/A9 did not affect RCAN3 Expression in normal or HSS-BMDMs (Supplement 18a, b). No significant differences in RCAN3 expression were observed in HSS-BMDMs from VEGFR1^+/-^ vs. VEGFR1^+/+^ mice (Supplement 18c). These data indicated that inhibiting VEGF_165_b decreases RCAN3 expression in HSS-ECs and HSS-BMDMs independent of VEGFR1-STAT3 or VEGFR1-S100A8/A9 signaling, respectively.

## Discussion

In our efforts to advance our understanding of the VEGF_165_b isoform in regulating the angiogenic response to hind limb ischemia, our study discovered a novel miR-17-20a-RCAN3 pathway that occurs specifically with the depletion of the anti-angiogenic VEGF_165_b in PAD. The ability of skeletal muscle to recover from ischemic damage that occurs experimentally from HLI and repeatedly during daily activity in patients with PAD is directly dependent on the extent of the angiogenic response in ischemic muscle^2^. To the best of our knowledge, our current study is the first to demonstrate a pathway by which removing the inhibitory effect of VEGF_165_b in ischemic muscle allows the expression of a truncated miR-17-20a cluster in ischemic ECs and Møs that targets RCAN3 to revascularize ischemic muscle in experimental PADs. The miR-17-92 cluster is a highly studied miRNA cluster in cancer research^25,26,28^. While studies, in general, are focused on the whole miR-17-92 cluster, in our study, we present specific roles of miR-17 and miR-20a within the miR-17-92 cluster that are regulated by VEGF_165_b inhibition. Although the first evidence of an oncogenic role (by promoting cell survival, proliferation, and angiogenesis) in B-cell lymphoma involved a truncated miR-17-92 cluster (without miR-92), hence the name ‘OncomiR’^24,49,50^ was given, two paralogs of the miR-17-92 cluster are already known to occur in humans: miR-106b-25 (miR-106b, -93, -25) and miR-106a-363 (miR-106a, -18b, -19b-2, -20b, -92a-2, and -363) clusters^51^.

The 15 miRNAs from these 3 paralogous clusters form four seed families that include the miR-17 family (miR-17, miR-20a, miR-106a, miR-20b, miR-106b, and miR-93); miR-18 family (miR-18a, miR-18b); miR-19 family (miR-19a, miR-19b-1, miR-19b-2) and miR-92 family (miR-92a, miR-92-a-2, miR-363, and miR-25). A previous study by Hazarika et al.^32^ from our group showed that miR-106b, miR-93, miR-106a, and miR-17 are among the top 10 differentially regulated genes between C57BL/6J vs. Balb/cJ ischemic muscle at day 3 post-HLI. Consistent with these data, in our current study, we observed miR-17 to be one of the significantly different miRNAs in the ischemic muscle of C57BL/6J and Balb/cJ mouse strains with pro-angiogenic properties in the ischemic environment. Our previous studies have also shown that miR-93 within the miR-106b-25 cluster exhibits pro-angiogenic properties in an ischemic environment^31,32^, and miR-106a within the miR-106a-363 cluster induces ischemic angiogenesis (unpublished data). Taken together, these studies further indicate that miRNAs within the miR-17 seed sequence family may play critical roles in regulating the pathology of PAD.

Limited information is available on the role of the miR-17-92 cluster members in cardiovascular diseases, including PAD. Hinkel et al.^27^ showed that inhibiting miR-92a improved functional recovery in PAD^27^. Landskroner-Eiger et al.^28^ have shown improved limb arteriogenesis in EC-specific miR-17-92 cluster-deficient mice in experimental PAD, and this study shows that specific targeting of Frizzled Class Receptor 4 (FZD4) and LDL Receptor Related Protein 6 (LRP6) by miR-19 within the cluster plays a causal role in decreasing blood flow recovery^28^. In contrast, a recent report by Chamorro-Jorganes et al.^52^ showed that EC-specific miR-17-92-deficient mice have blunted physiological retinal angiogenesis as well as diminished VEGF-induced angiogenesis^52^. Furthermore, deletion of the miR-17-92 cluster in renal proximal tubules or in ECs resulted in severe renal dysfunction and promoted microvascular rarefaction^53,54^. In our current study, increased expression of the miR-17-20a cluster by VEGF_165_b inhibition enhanced perfusion recovery. These data indicate that the phenotypic effect observed in gene knockout models is not the same as fine-tuning^55^ a miRNA level/function. This can be reflected in the lack of angiogenesis in miR-17-92 gene knockout models^28^ vs. increased ischemic-muscle revascularization when the miR-17-20a levels/function are fine-tuned (similar to their expression in normal conditions) by VEGF_165_b inhibition. Taken together, these data indicate that VEGF_165_b inhibition fine-tunes the expression of the miR-17-20a cluster, which is sufficient to induce ischemic-muscle revascularization in PAD.

Interestingly, Chamorro-Jorganes et al.^52^ described that VEGF induces miR-17-92 expression via ELK1 (extracellular signal-regulated kinase (ERK)-ETS-like-1), suggesting a potential role of VEGFR2 signaling in regulating the miR-17-92 cluster^52^. However, our recent reports have shown that VEGF_165_b induces VEGFR2 activation and that inhibiting VEGF_165_b decreases VEGFR2 activation. We anticipated that VEGF_165_b inhibition would induce the miR-17-20a-RCAN3 pathway dependent on VEGFR1-STAT3 (in ischemic ECs) or VEGFR1-S100A8/A9 (in ischemic Møs). However, our data showed that inhibiting VEGF_165_b regulates the miR-17-20-RCAN3 pathway independent of VEGFR1-STAT3 or VEGFR1-S100A8/A9 signaling in ischemic ECs and ischemic Møs, respectively. While a full explanation of the involvement of alternative signaling pathways regulated by VEGF_165_b to control miR-17-20a expression is beyond the scope of this report, these data suggest the possibility of direct epigenetic regulation by VEGF_165_b isoforms in ischemic vasculature. In support of this, previous reports have shown that VEGF-A accumulates in the nucleus during wound healing and in response to hypoxia^56,57^. Since VEGF_165_a and VEGF_165_b isoforms only differ in exon 8, further studies to determine the role of VEGF_165_b in nuclear vs. membrane compartments will present evidence for novel epigenetic vs. receptor-mediated signaling mechanisms downstream of VEGF_165_b inhibition that regulate miR-17-20a cluster expression in ways different from VEGFR2 or our previously published VEGFR1 signaling in ischemic vasculature.

Distinct functions and expression of the specific miRs within this cluster have been well established in several tumor studies^58–67^. Reports on the 6 miRs that comprise the miR-17-92 cluster have reported differential expression patterns. For example, miR-92 is expressed at much higher levels than the rest of the cluster members in several tumors, including glioma, colorectal cancer, and breast cancer^58,61,62^. Accordingly, in our current study, we consistently observed lower miR-18 expression than other cluster members in primary skeletal muscle, ECs, and BMDMs under normal or ischemic conditions. Although the miR-17-92 cluster is conserved between humans and mice, it is important to note a relatively lower expression (or lack of) of miR-18 expression in primary mouse ECs but not in human ECs in our study. This suggests a more complex evolutionary regulation of this cluster across species, and distinct context-dependent mechanisms operate in biogenesis, processing, and/or degradation within the members of this miRNA cluster^68,69^.

Our current study identified RCAN3 as a novel regulator of PAD. While HSS induced RCAN3 expression in SkMVECs, no significant difference in RCAN3 expression was observed in HSS-HUVECs vs. normoxic controls. Nevertheless, RCAN3 overexpression inhibited HUVEC angiogenic capacity, and silencing RCAN3 enhanced SkMVEC angiogenic capacity, indicating a functional role of the miR-17-20a-RCAN3 pathway in regulating ischemic angiogenesis. Limited information is available on the pathological roles of RCAN3. Recent studies have shown that RCAN3 decreases arthritis development in collagen-induced murine models^70^, and overexpressing RCAN3 or RCAN3-derived peptide has been shown to inhibit tumor progression^29^. Furthermore, RCAN3 has been shown to induce antiproliferative effects in HUVECs^30^. Our data showing that transcriptional and translational repression of RCAN3 by miR-17 and miR-20a enhances perfusion recovery in preclinical PAD models presents RCAN3 as a putative miR-17 and miR-20a target that regulates perfusion recovery in PAD. While further studies are needed to determine whether sex plays a role in regulating the miR-17-20a-RCAN3 pathway in experimental PAD, our study is the first report identifying RCAN3 as a downstream regulator of VEGF_165_b. The ability of RCAN3 to regulate ischemic EC angiogenic capacity and Mø polarization makes it an attractive PAD therapeutic.

## Conclusions

We present new evidence that removal of VEGF_165_b is necessary to induce miR-17 and miR-20a expression that revascularizes ischemic muscle by targeting RCAN3, a novel regulator of PAD. Further studies are needed to understand the molecular mechanisms that regulate the transcriptional control of miR-17-92 cluster members that result in their distinct expression pattern post VEGF_165_b inhibition. Advances in miRNA therapeutics and monoclonal antibody-based therapeutics point toward the potential to target miR-17-20a and RCAN3 for clinical applications.

### Supplementary information

Description of Additional Supplementary Files

Supplementary Information

Supplementary Data 1

Reporting Summary

## Data Availability

All data generated or analyzed during this study are included in this article as Source data. Source data for Figs. 1–6, Supplementary figs. 2–11, 14, 16–18, and the list of major reagents used in the study can be found in Supplementary Data 1. All other data are available from the corresponding author (or other sources, as applicable) on reasonable request.
