# Supplementary material for: Inhibiting anti-angiogenic VEGF165b activates a miR-17-20a-Calcipressin-3 pathway that revascularizes ischemic muscle in peripheral artery disease
Source: Commun Med (Lond). 2024 Jan 5;4:3. doi: 10.1038/s43856-023-00431-5 (PMC10770062; doi:10.1038/s43856-023-00431-5)
Supplement: Supplementary file 1 — Description of Additional Supplementary Files [file 43856_2023_431_MOESM1_ESM.docx]

**Description of Additional Supplementary Files**

**File Name:** Supplementary Data 1

**Description:** Source data for Figures 1-6, Supplementary figures 2-11, 14, 16-18 and major reagents.
