# Supplementary material for: Inhibiting anti-angiogenic VEGF165b activates a miR-17-20a-Calcipressin-3 pathway that revascularizes ischemic muscle in peripheral artery disease
Source: Commun Med (Lond). 2024 Jan 5;4:3. doi: 10.1038/s43856-023-00431-5 (PMC10770062; doi:10.1038/s43856-023-00431-5)
Supplement: Supplementary file 2 — Supplementary Information [file 43856_2023_431_MOESM2_ESM.pdf]

Supplementary Fig-1: Endothelial marker expression in primary Skeletal muscle microvascular ECs

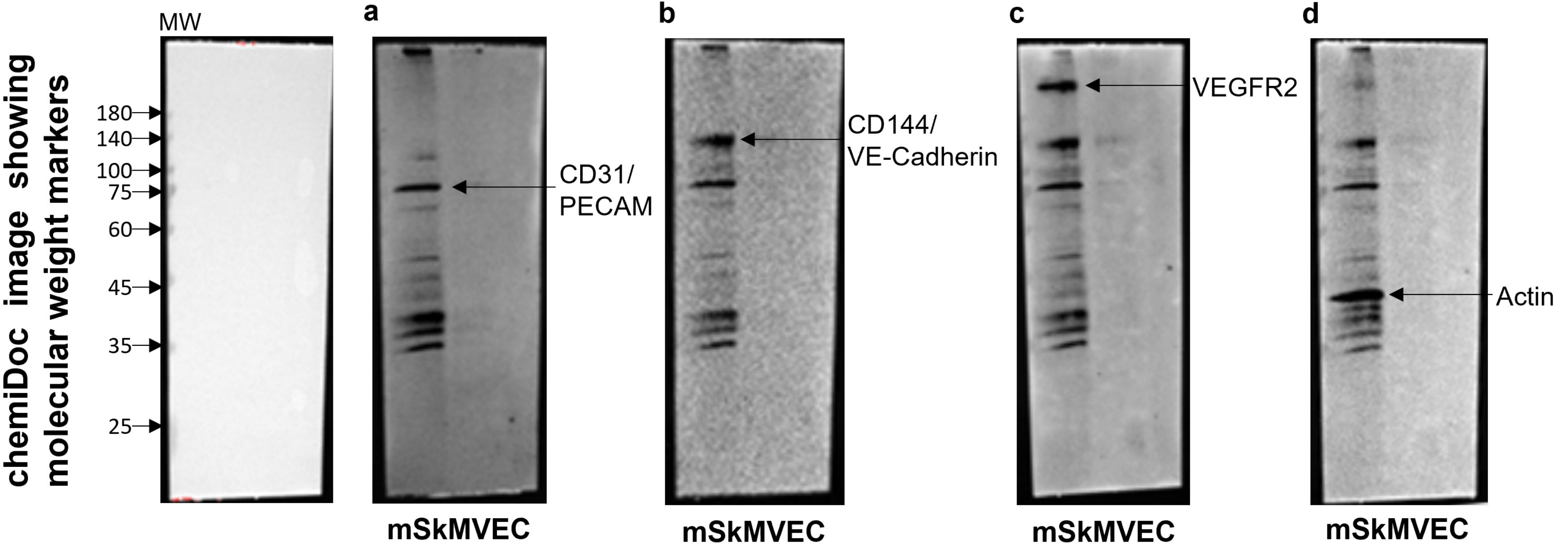

Western blot analysis of a) CD31/PECAM1, b) CD144/VE-Cadherin, c) VEGFR2 and d) Actin in SkMVECs isolated from mouse gastrocnemius and tibialis anterior at passage-2. Since the molecular weight markers were not clearly visible on the membranes, ChemiDoc image was presented separately to show molecular weight markers.

Supplementary Fig-2: VEGF$_{165}$b-inhibition restores the expression of miR-17-92 cluster in HSS HUVECs comparable to normal HUVECs.

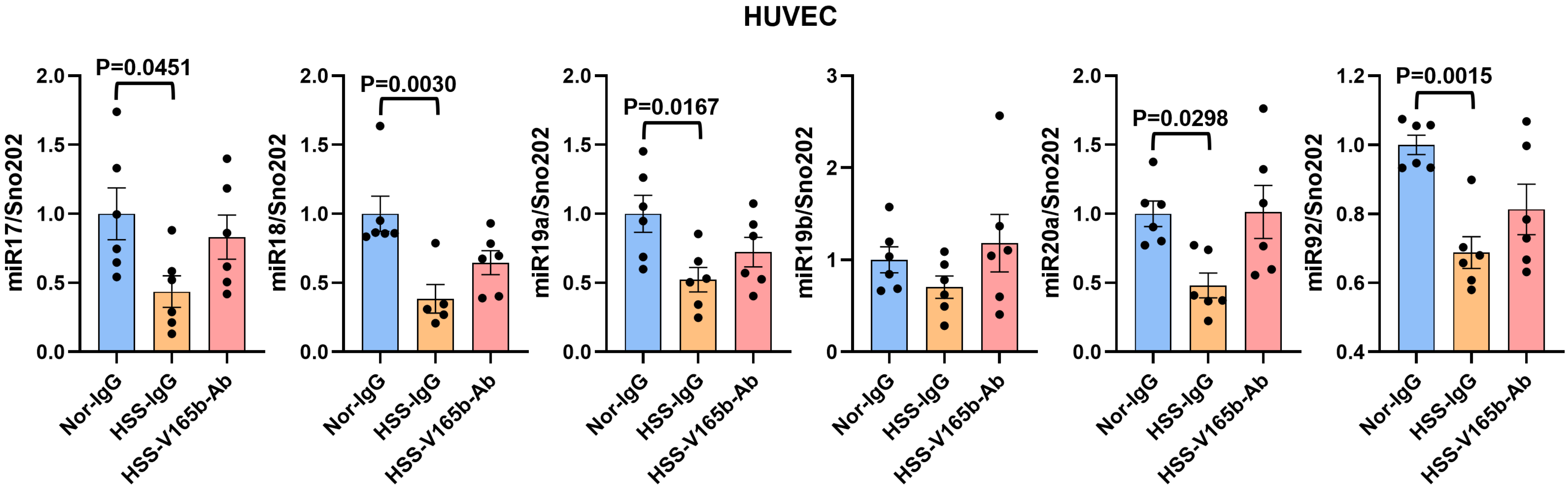

PCR analysis of mi-R17-92 cluster expression in Human Umbilical Vein Endothelial Cells (HUVECs) under normal (Nor, blue bars) or HSS conditions treated with IgG (yellow bars) or VEGF$_{165}$b-Ab (V$_{165}$b-Ab, pink bars) for 24h. n=6. One Way ANOVA with Bonferroni's multiple comparison. P<0.05 considered significant. Data from the biological replicates are presented as Mean ± Standard Error.

Supplementary Fig-3: $VEGF_{165}$b-inhibition induces miR-17-20a cluster expression in C57BL/6 mice.

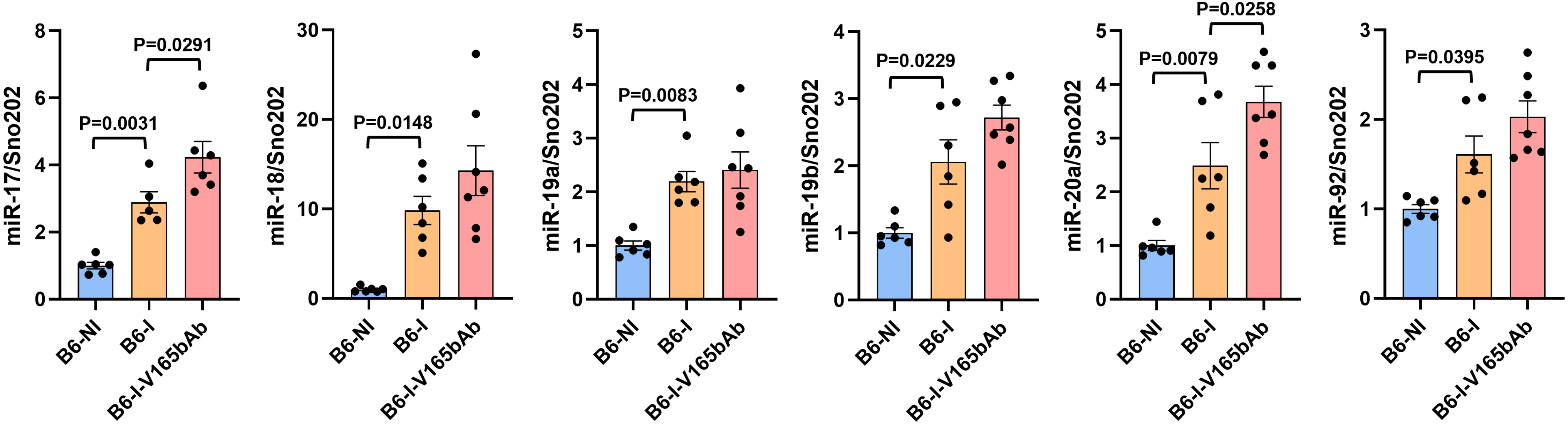

qPCR of miR-17-92 cluster in C57BL/6J (B6) non-ischemic (NI, n=6, blue bars) muscle and ischemic muscle (I, n=6) treated with IgG (yellow bars) or $VEGF_{165}$b-Ab (n=7, pink bars) at day-3 post-HLI. One Way ANOVA with Bonferroni select pair comparison for miR-17, miR-18, miR-19a, miR-20a and miR-92. Brown-Forsythe and Welch ANOVA test with Dunnett's T3 multiple comparison for miR-19b. P<0.05 considered significant Data from the biological replicates are presented as Mean ± Standard Error.

Supplementary Fig-4: STAT3 inhibition did not affect the expression of miR-17-20a in ischemic ECs.

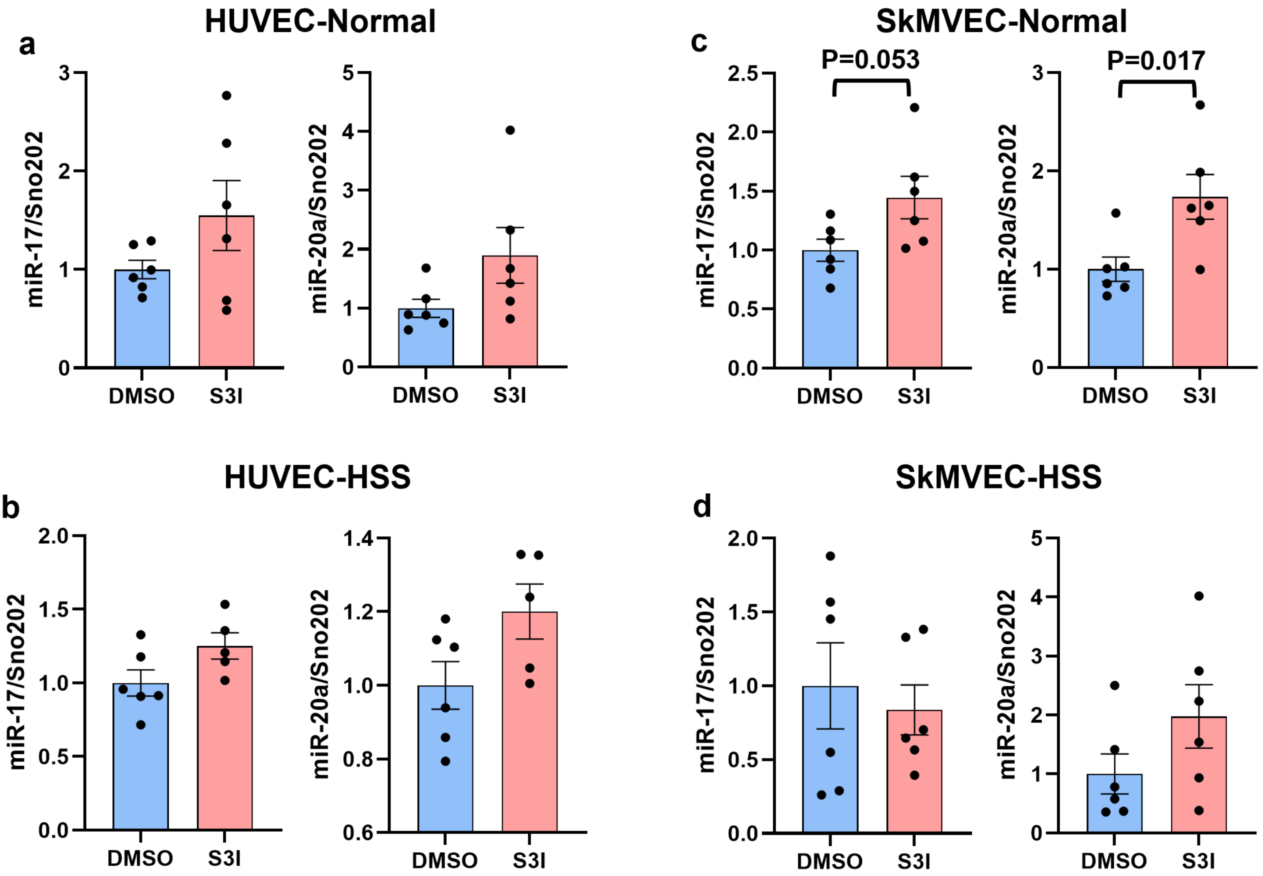

qPCR of miR-17-20a expression in a) normal HUVECs (n=6), b) HSS-HUVECs (n=5), c) Normal SkMVECs (n=6) and, d) HSS SkMVECs (n=6) treated with DMSO (blue bars) or S3I201 (pink bars, 100 μM) for 24h under normal or HSS conditions. Unpaired T-Test. P<0.05 considered significant. Data from the biological replicates are presented as Mean ± Standard Error.

Supplementary Fig-5: VEGFR1$^{+/-}$ SkMVECs have ~50% decrease in VEGFR1 expression compared to littermate controls in ischemic conditions.

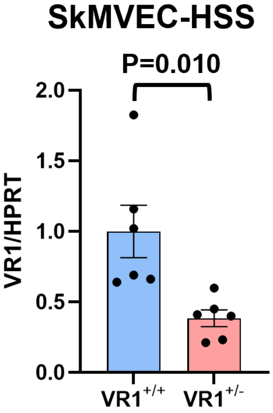

qPCR of VEGFR1 expression in SkMVECs isolated from VEGFR1$^{+/+}$ (VR1$^{+/+}$, blue bar) or VEGFR$^{+/-}$ (VR1$^{+/-}$, pink bar) mice challenged with HSS, n=6. Unpaired T-Test. $P<0.05$ considered significant. Data from the biological replicates are presented as Mean ± Standard Error.

Supplementary Fig-6: VEGFR1 deficiency did not affect miR-17-20a expression in ischemic SkMVECs.

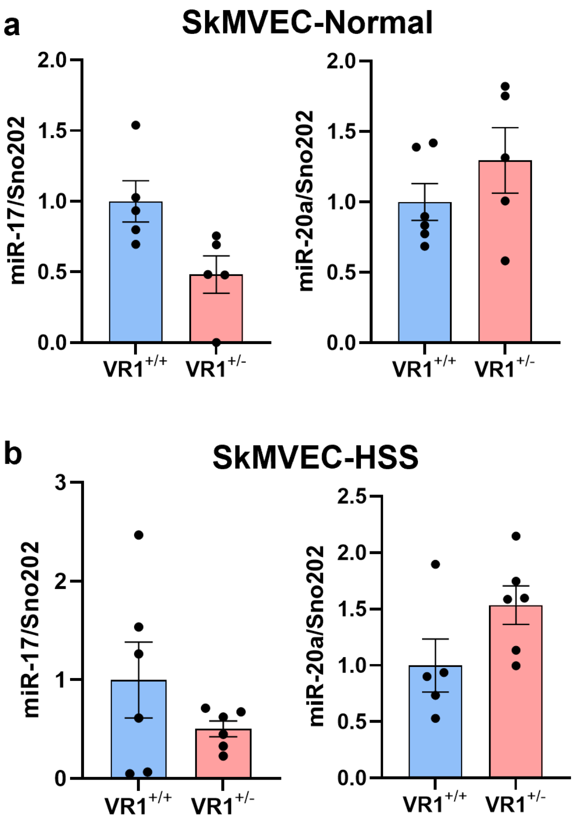

qPCR of miR-17 and miR-20a expression in a) Normal (n=6), b) HSS (n=6) challenged SkMVECs isolated from VEGFR1$^{+/+}$ (VR1$^{+/+}$, blue bars) or VEGFR$^{+/-}$ (VR1$^{+/-}$, pink bars) mice. Unpaired T-Test with Welch's correction for miR-17 in HSS conditions. Unpaired T-Test for remaining comparisons. Outliers removed by Grubb's test. $P<0.05$ considered significant. Data from the biological replicates are presented as Mean ± Standard Error.

Supplementary Fig-7: Silencing S100A8/A9 did not affect the expression of miR-17-20a in ischemic Møs.

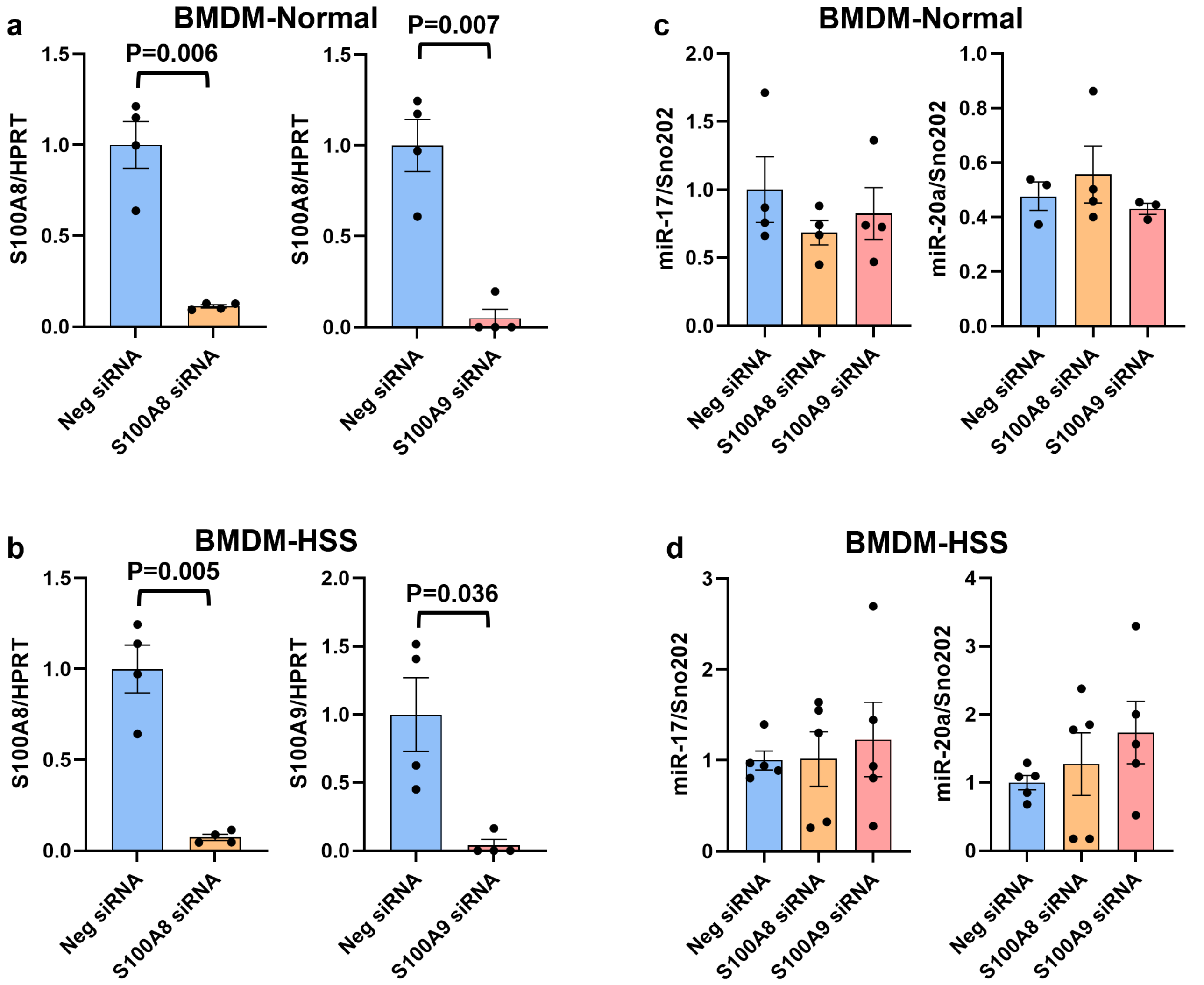

qPCR of S100A8 and S100A9 in a) normal BMDMs transfected with control siRNA (Neg siRNA, blue bars), S100A8 (yellow bars) or S100A9 (pink bars) silencing RNA, n=4, Unpaired T-Test with Welch's correction for S100A8, Unpaired T-Test for S100A9, b) HSS BMDMs transfected with control siRNA (Neg siRNA, blue bars), S100A8 (yellow bars) or S100A9 (pink bars) silencing RNA, n=4, Unpaired T-Test with Welch's correction. qPCR analysis of miR-17 and miR-20a in c) normal BMDMs transfected with control siRNA (Neg siRNA, blue bars), S100A8 (yellow bars) or S100A9 (yellow bars) silencing RNA, n=4, One WAY ANOVA with Bonferroni select pair comparison, d) HSS BMDMs transfected with control siRNA (Neg siRNA, blue bars), S100A8 (yellow bars) or S100A9 (pink bars) silencing RNA, n=5, One WAY ANOVA with Bonferroni select pair comparison. P<0.05 considered significant. Data from the biological replicates are presented as Mean ± Standard Error.

Supplementary Fig-8: VEGFR1 deficiency did not affect miR-17-20a expression in ischemic Møs.

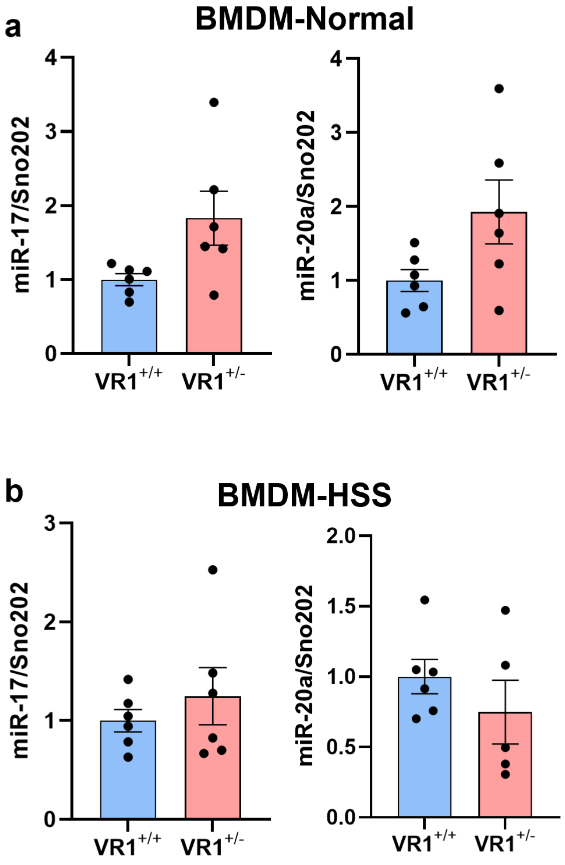

qPCR of miR-17 and miR-20a expression in a) Normal (n=6), b) HSS (n=6) challenged BMDMs isolated from VEGFR1$^{+/+}$ (VR1$^{+/+}$, blue bars) or VEGFR$^{+/-}$ (VR1$^{+/-}$, pink bars) mice. Unpaired T-Test with Welch's correction for miR-17 and miR-20a in normal conditions. Unpaired T-Test for HSS conditions. $P<0.05$ considered significant. Data from the biological replicates are presented as Mean ± Standard Error.

Supplementary Fig-9: qPCR analysis to confirm miR-17-20a inhibition in C57BL/6J ischemic muscle.

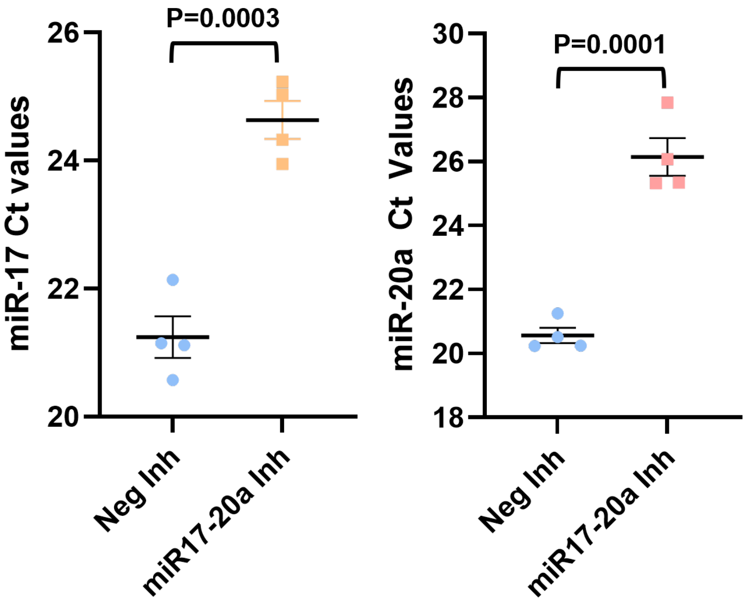

qPCR analysis (Ct Values) of miR-17 and miR-20a in C57BL/6J mice skeletal muscle treated with negative inhibitor (Neg Inh, blue circles) or miR17-20a inhibitor (a combination of miR-17 inhibitor (yellow squares) and miR-20a inhibitor (pink squares)) immediately after hind limb ischemia and assayed at day-3 post-hind limb ischemia. n=4. Unpaired T-test. $P<0.05$ considered significant. Data from the biological replicates are presented as Mean ± Standard Error.

Supplementary Fig-10: qPCR analysis to confirm miR-17-20a inhibition or overexpression in HUVECs.

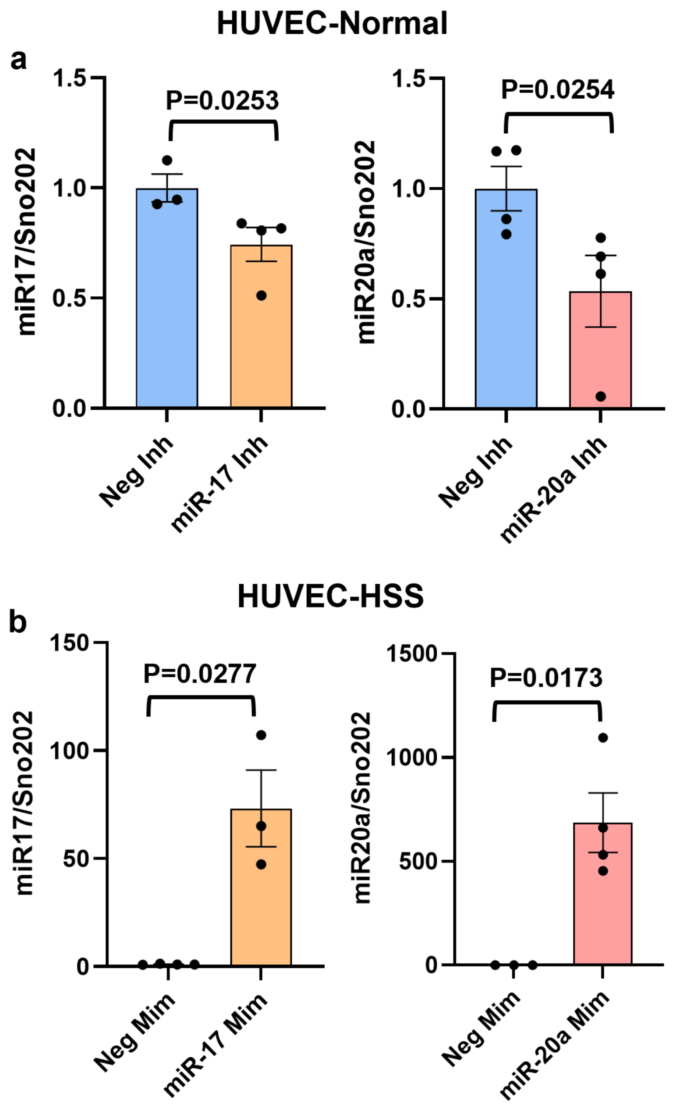

qPCR analysis of miR-17 and miR-20a expression in a) HUVECs transfected with negative inhibitor (Neg Inh, n=3, blue bars), miR-17 inhibitor (miR-17 Inh, n=4, yellow bars, Unpaired T-test with Welch's correction) or miR-20a inhibitor (miR-20a Inh, pink bars, Unpaired T-test) for 24hr under normal growth conditions. $P<0.05$ considered significant. b) HUVECs transfected with negative mimic (Neg Mim, n=3, blue bars), miR-17 mimic (miR-17 Mim, n=4, yellow bars, Unpaired T-test with Welch's correction) or miR-20a mimic (miR-20a Mim, pink bars, Unpaired T-test with Welch's correction) for 24hr under normal growth conditions followed by HSS for 24h. $P<0.05$ considered significant. Data from the biological replicates are presented as Mean ± Standard Error.

Supplementary Fig-11: qPCR analysis to confirm miR-17-20a inhibition or overexpression in BMDMs.

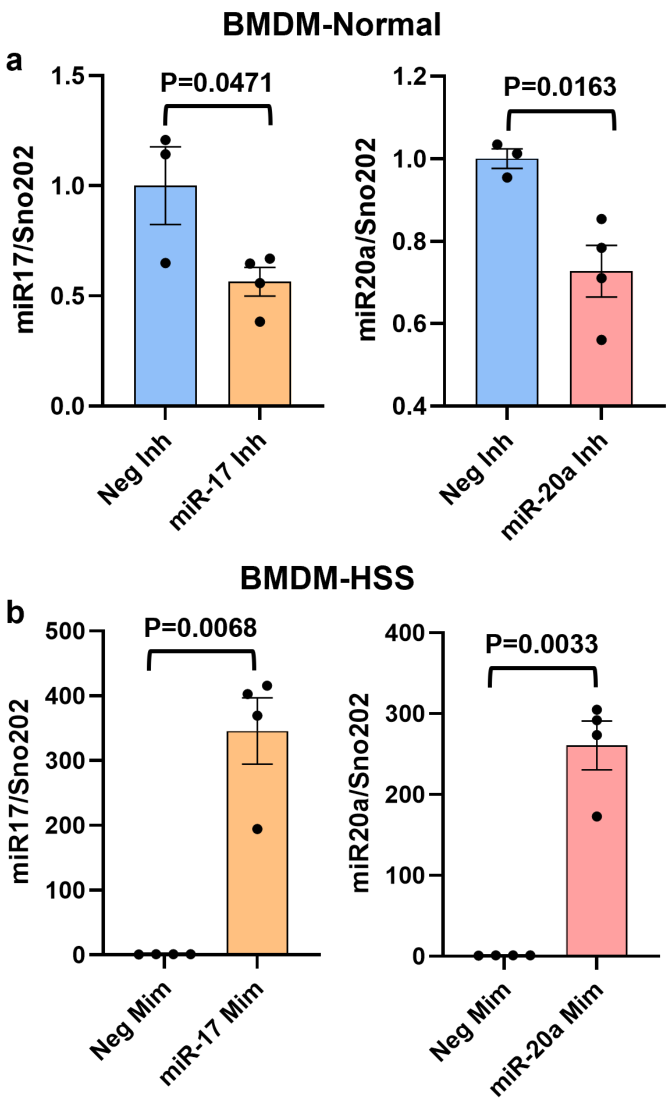

qPCR analysis of miR-17 and miR-20a expression in a) Balb/cJ BMDMs transfected with negative inhibitor (Neg Inh, blue bars), miR-17 inhibitor (miR-17 Inh, yellow bars, Unpaired T-Test) or miR-20a inhibitor (miR-20a Inh, pink bars, Unpaired T-Test) for 24hr under normal growth conditions. n=4. P<0.05 considered significant. b) Balb/cJ BMDMs transfected with negative mimic (Neg Mim, blue bars), miR-17 Mimic (miR-17 Mim, yellow bars, Unpaired T-Test with Welch's correction) or miR-20a Mimic (miR-20a Mim, pink bars, Unpaired T-Test with Welch's correction) for 24hr under normal growth conditions followed by HSS for 6h. n=4. P<0.05 considered significant. Data from the biological replicates are presented as Mean ± Standard Error.

Supplementary Fig-12: Western blot analysis to confirm equal pulldown of Argonaute-2.

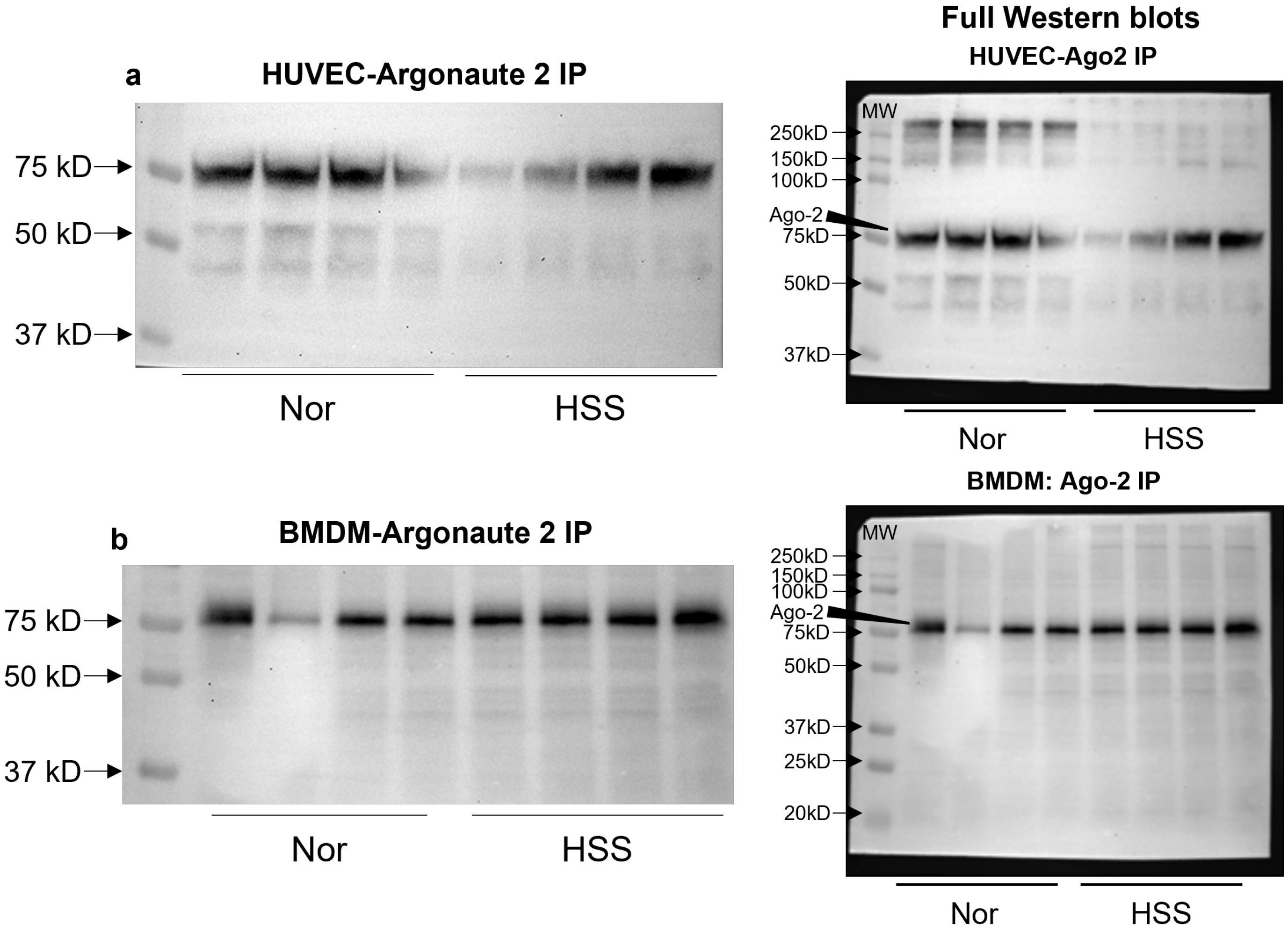

Western blot analysis of Argonaute-2 in Argonaute-2 immunoprecipitated samples showing equal pull down in normal vs. HSS challenged a) HUVECs and b) BMDMs. n=4.

Supplementary Fig-13: Inhibiting miR-17-20a expression increase RCAN3 levels in ischemic muscle.

Full Western blot for Fig-5a

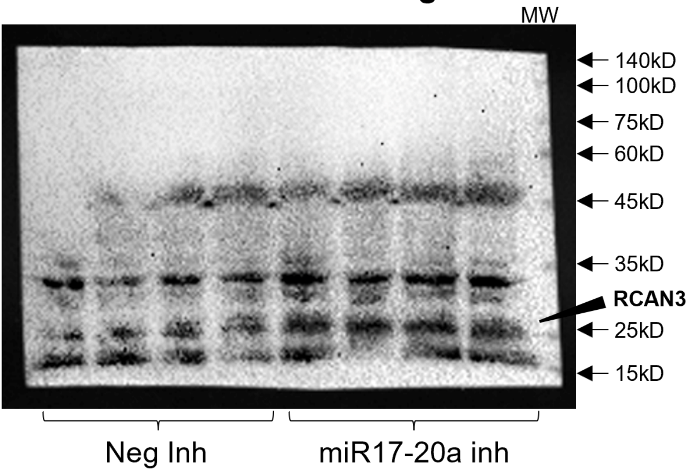

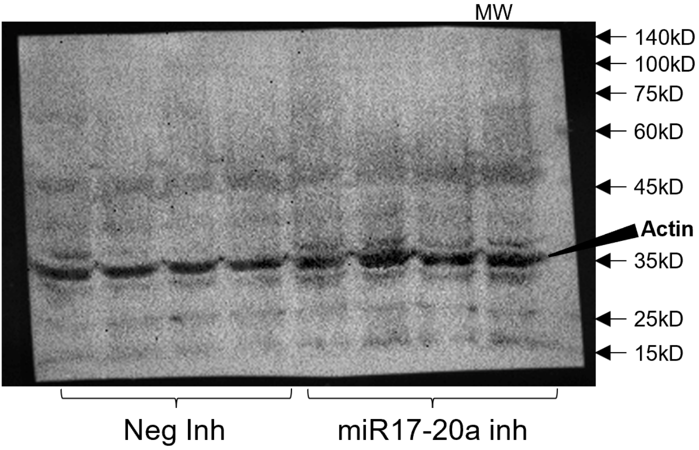

Full length western blots of RCAN3 and Actin in ischemic muscle treated with Neg Inh or a combination of miR-17 and miR-20a inhibitor (miR-17-20 Inh). n=4.

Supplementary Fig-14: qPCR analysis to confirm RCAN3 overexpression in C57BL/6J ischemic muscle.

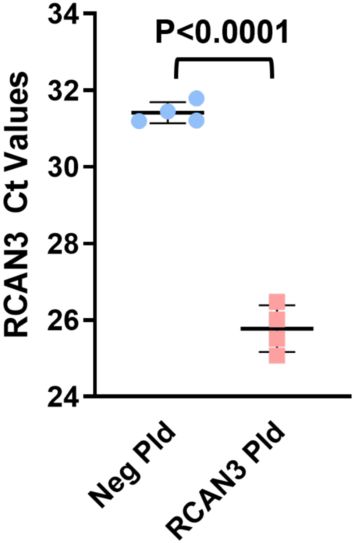

qPCR analysis of RCAN3 Ct values in C57BL/6J mice treated with control plasmid (Neg Pld, blue circles) or RCAN3 expressing plasmid (RCAN3 Pld, pink squares) at day-3 post-hind limb ischemia, n=4, Unpaired T-test. $P<0.05$ considered significant. Data from the biological replicates are presented as Mean ± Standard Error.

Supplementary Fig-15: RCAN3 levels in normal and HSS challenged BMDMs, SkMVECs and HUVECs.

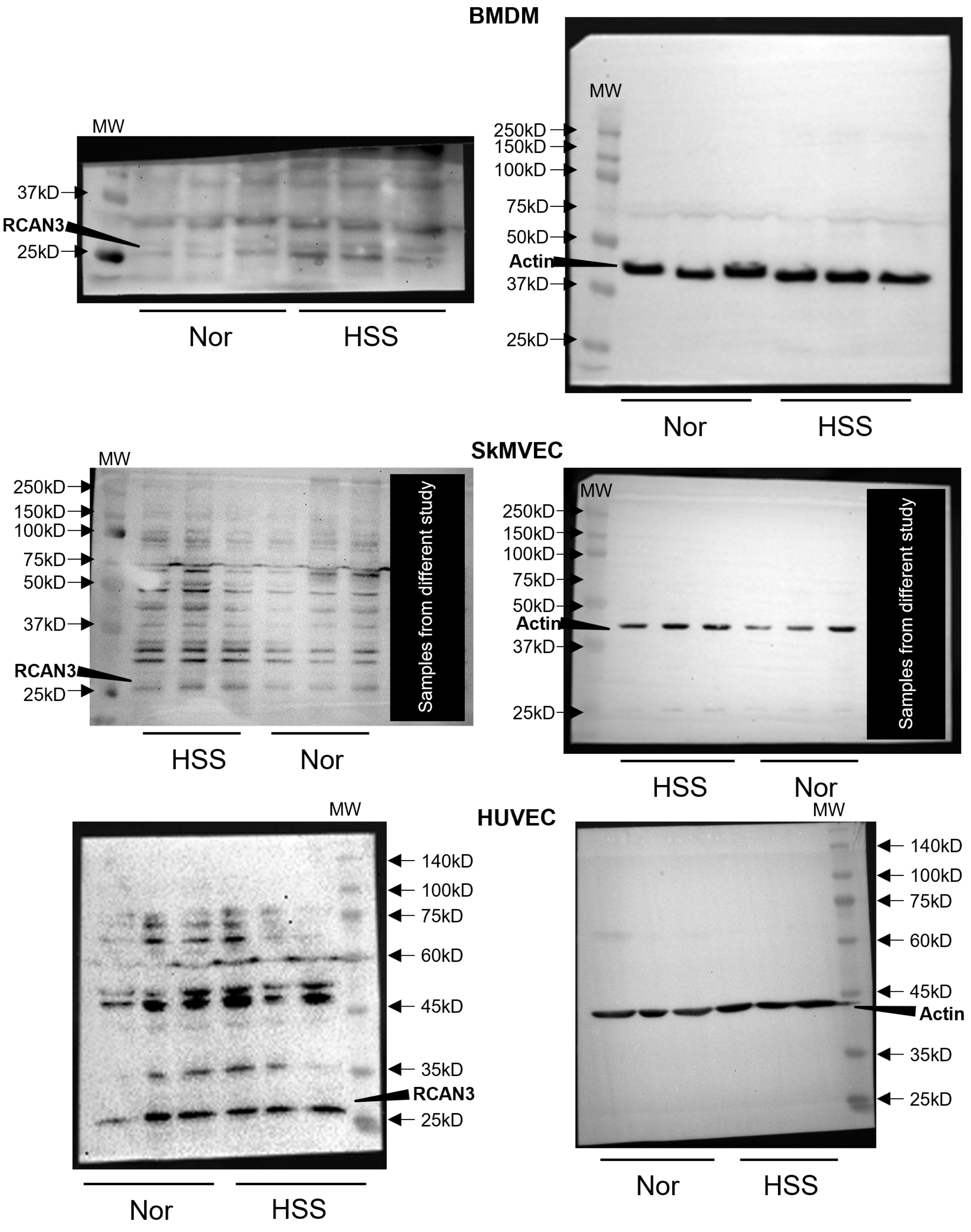

Full length western blots of RCAN3 and Actin presented in Fig-6a-c. n=3.

Supplementary Fig-16: qPCR analysis to confirm RCAN3 overexpression in HUVECs and BMDMs.

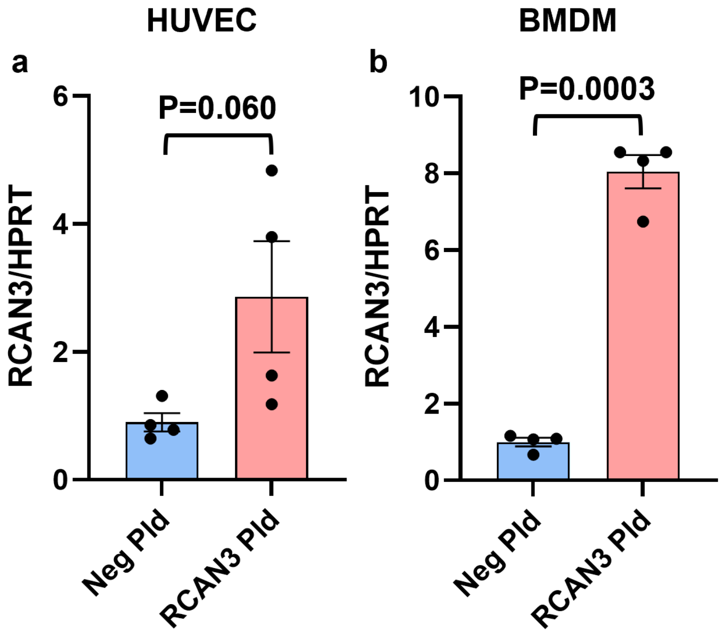

qPCR analysis of RCAN3 expression in a) HUVECs, b) BMDMs transfected with control (Neg Pld, blue bars) or RCAN3 expressing plasmid (RCAN3 Pld, pink bars). n=4. Unpaired T-test with Welch's correction. $P<0.05$ considered significant. Data from the biological replicates are presented as Mean ± Standard Error.

Supplementary Fig-17: STAT3 inhibition or VEGFR1 deficiency did not affect RCAN3 expression in ischemic ECs.

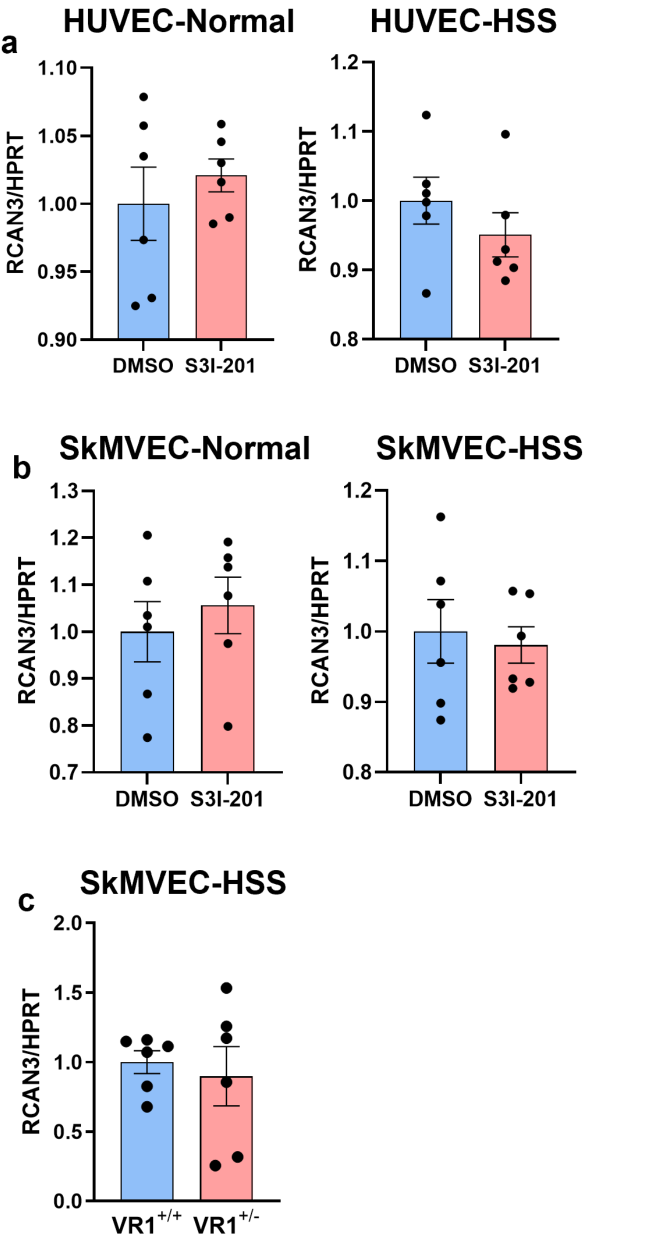

qPCR of RCAN3 expression in a) normal or HSS challenged HUVECs treated with DMSO (blue bars) or S31-201 (pink bars) for 24h, n=6, Unpaired T-Test, b) normal or HSS challenged SkMVECs treated with DMSO (blue bars) or S31-201 (pink bars) for 24h, n=6, Unpaired T-Test, c) HSS challenged SkMVECs isolated from VEGFR1$^{+/+}$ (VR1$^{+/+}$, blue bars) or VEGFR$^{+/-}$ (VR1$^{+/-}$, pink bars) mice, n=6, Unpaired T-test with Welch's correction. P<0.05 considered significant. Data from the biological replicates are presented as Mean ± Standard Error.

Supplementary Fig-18: Silencing S100A8/A9 or VEGFR1 deficiency did not affect RCAN3 expression in ischemic Møs.

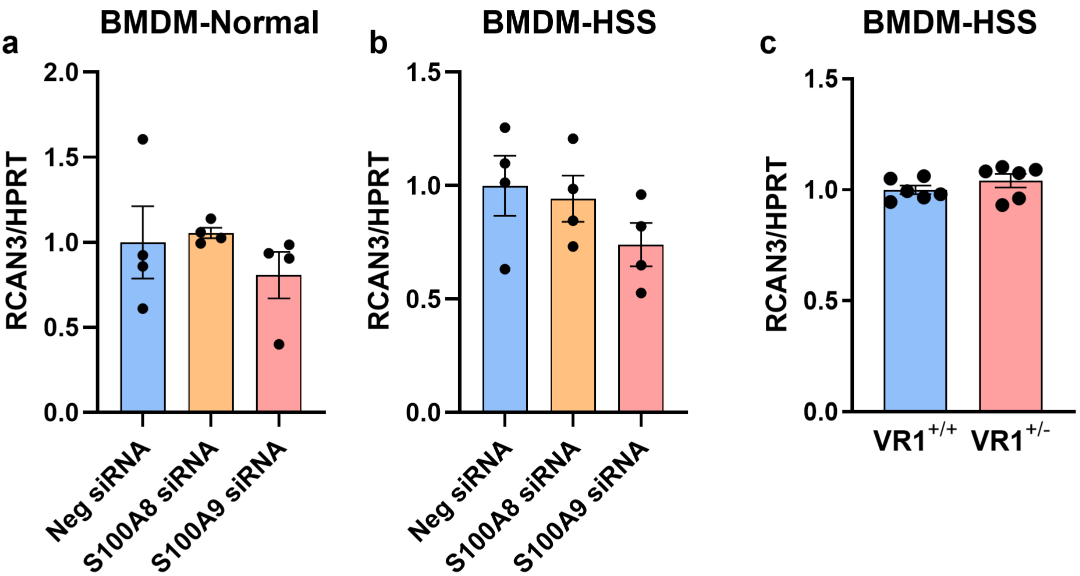

qPCR of RCAN3 expression in a) normal BMDMs transfected with control siRNA (Neg siRNA, blue bars), S100A8 (yellow bars) or S100A9 (pink bars) silencing RNA, n=4, One Way ANOVA with Bonferroni select pair comparison, b) HSS BMDMs transfected with control siRNA (Neg siRNA, blue bars), S100A8 (yellow bars) or S100A9 (pink bars) silencing RNA, n=4, One Way ANOVA with Bonferroni select pair comparison, c) HSS challenged BMDMs isolated from VEGFR1$^{+/+}$ (VR1$^{+/+}$, blue bars) or VEGFR$^{+/-}$ (VR1$^{+/-}$, pink bars) mice, n=5, Unpaired T-Test. $P<0.05$ considered significant. Data from the biological replicates are presented as Mean ± Standard Error.
