# Supplementary material for: Inhibiting anti-angiogenic VEGF165b activates a miR-17-20a-Calcipressin-3 pathway that revascularizes ischemic muscle in peripheral artery disease
Source: Commun Med (Lond). 2024 Jan 5;4:3. doi: 10.1038/s43856-023-00431-5 (PMC10770062; doi:10.1038/s43856-023-00431-5)
Supplement: Supplementary file 4 — Reporting Summary [file 43856_2023_431_MOESM4_ESM.pdf]

nature portfolio

Corresponding author(s): Vijay Ganta

Last updated by author(s): Oct-18-23

Reporting Summary

Nature Portfolio wishes to improve the reproducibility of the work that we publish. This form provides structure for consistency and transparency in reporting. For further information on Nature Portfolio policies, see our Editorial Policies and the Editorial Policy Checklist.

Statistics

For all statistical analyses, confirm that the following items are present in the figure legend, table legend, main text, or Methods section.

n/a	Confirmed	
☐	☒	The exact sample size (*n*) for each experimental group/condition, given as a discrete number and unit of measurement
☐	☒	A statement on whether measurements were taken from distinct samples or whether the same sample was measured repeatedly
☐	☒	The statistical test(s) used AND whether they are one- or two-sided<br>*Only common tests should be described solely by name; describe more complex techniques in the Methods section.*
☐	☒	A description of all covariates tested
☐	☒	A description of any assumptions or corrections, such as tests of normality and adjustment for multiple comparisons
☐	☒	A full description of the statistical parameters including central tendency (e.g. means) or other basic estimates (e.g. regression coefficient) AND variation (e.g. standard deviation) or associated estimates of uncertainty (e.g. confidence intervals)
☐	☒	For null hypothesis testing, the test statistic (e.g. *F*, *t*, *r*) with confidence intervals, effect sizes, degrees of freedom and *P* value noted<br>*Give P values as exact values whenever suitable.*
☒	☐	For Bayesian analysis, information on the choice of priors and Markov chain Monte Carlo settings
☒	☐	For hierarchical and complex designs, identification of the appropriate level for tests and full reporting of outcomes
☒	☐	Estimates of effect sizes (e.g. Cohen's *d*, Pearson's *r*), indicating how they were calculated

Our web collection on statistics for biologists contains articles on many of the points above.

Software and code

Policy information about availability of computer code

Data collection	No custom Software used
Data analysis	No custom Software used

For manuscripts utilizing custom algorithms or software that are central to the research but not yet described in published literature, software must be made available to editors and reviewers. We strongly encourage code deposition in a community repository (e.g. GitHub). See the Nature Portfolio guidelines for submitting code & software for further information.

Data

Policy information about availability of data

All manuscripts must include a data availability statement. This statement should provide the following information, where applicable:
- Accession codes, unique identifiers, or web links for publicly available datasets
- A description of any restrictions on data availability
- For clinical datasets or third party data, please ensure that the statement adheres to our policy

Data will be provided upon reasonable request

Human research participants

Policy information about studies involving human research participants and Sex and Gender in Research.

Reporting on sex and gender	Not applicable
Population characteristics	Not applicable
Recruitment	Not applicable
Ethics oversight	Not applicable

Note that full information on the approval of the study protocol must also be provided in the manuscript.

Field-specific reporting

Please select the one below that is the best fit for your research. If you are not sure, read the appropriate sections before making your selection.

☒ Life sciences ☐ Behavioural & social sciences ☐ Ecological, evolutionary & environmental sciences

For a reference copy of the document with all sections, see nature.com/documents/nr-reporting-summary-flat.pdf

Life sciences study design

All studies must disclose on these points even when the disclosure is negative.

Sample size	Sample size was based upon our previous studies where an a priori power analysis by Kruskal-Wallis test showed that with a minimum n=4-5/group, we will have 80% power if a pro-angiogenic treatment will have zero necrosis vs. grade-1 necrosis without treatment Based on this sample size estimate, we had at least n=4 per group in our current study.
Data exclusions	Grubb's Outlier test was performed to detect any outliers. Detected outliers were excluded from the data analysis. No data was excluded without confirmation by Grubb's Outlier test
Replication	In vitro experiments were repeated twice with a minimum of 3 replicates
Randomization	Mice were randomly allocated to groups
Blinding	Laser scanning to measure blood flow and microvascular density measurements were performed by the surgeon followed by tissue collection, immunostaining and quantification by personnel blinded to the experimental groups

Reporting for specific materials, systems and methods

We require information from authors about some types of materials, experimental systems and methods used in many studies. Here, indicate whether each material, system or method listed is relevant to your study. If you are not sure if a list item applies to your research, read the appropriate section before selecting a response.

Materials & experimental systems

n/a	Involved in the study	
☐	☒	Antibodies
☐	☒	Eukaryotic cell lines
☒	☐	Palaeontology and archaeology
☐	☒	Animals and other organisms
☒	☐	Clinical data
☒	☐	Dual use research of concern

Methods

n/a	Involved in the study	
☒	☐	ChIP-seq
☒	☐	Flow cytometry
☒	☐	MRI-based neuroimaging

Antibodies

Antibodies used

1. Anti Rabbit Argonaute-2 monoclonal Antibody (Clone: EPR10411, Knockout validated), Abcam, Catalog No: ab186733
2. Anti Mouse Calcipressin 3 (RCAN3) monoclonal Antibody [Clone ID: OTI1B2], Origene, Catalog No: TA505496
3. Anti Mouse VEGF165b monoclonal Antibody, R&D, Catalog No: MAB3045
4. Anti Rat CD31 monoclonal Antibody (Clone MEC 13.3 for cell isolation), BD, Catalog No: 553370

5. Anti Armenian Hamster CD31 monoclonal Antibody (2H8) (Immunohistochemistry), ThermoFisher, Catalog No: MA3105
6. Anti Mouse α-Actin monoclonal Antibody (alpha-SM1), Santa Cruz, Catalog No: sc-130617
7. Anti Mouse PCNA (Ab-1) monoclonal Antibody (Clone: PC10), Millipore-Sigma, Catalog No: NA03
8. Anti Rabbit Actin polyclonal Antibody, Sigma, Catalog No: A2103
9. Anti Rabbit VE-Cadherin polyclonal antibody, Cayman Chemicals, Catalog No: 160840
10. Anti Rabbit VEGFR2 monoclonal antibody, Cell Signaling and Technology, Catalog No: 2479,

Validation

1. Rabbit monoclonal [EPR10411] to Argonaute-2 (Knockout validated), Abcam, ab186733: Used to immunoprecipitate Ago-2 in several publications, recently from Zhang et al (PMID: 34853466) and Murmann et al (PMID: 36585400)
2. RCalcipressin 3 (RCAN3) Mouse Monoclonal Antibody [Clone ID: OTI1B2], Origene, TA505496: Antibody was validated by western blot analysis of HEK cells expressing RCAN3. A single band was shown in the data sheet and on the web page. https://www.origene.com/catalog/antibodies/primary-antibodies/ta505496/calcipressin-3-rcan3-mouse-monoclonal-antibody-clone-id-oti1b2
3. VEGF165b Antibody, R&D, MAB3045: This antibody has been used and validated in our previous publications. PMID: 30586702, PMID: 36078086, PMID: 27974423
4. CD31 Antibody (cell isolation), BD, 553370: This antibody has been used to isolate endothelial cells from mouse tissues in the following publications. PMID: 30728372, PMID: 34950893
5. CD31 Antibody (Immunohistochemistry), ThermoFisher, MA3105: This antibody has been used and validated in our previous publication. PMID: 36078086
6. α-Actin Antibody (alpha-SM1), Santa Cruz, sc-130617; This antiboyd has been used and validated in the publication from Chen et al., (PMID: 36658107)
7. Anti-PCNA (Ab-1) Mouse mAb (PC10), Millipore-Sigma, NA03: This antibody has been used and validated in the publication from Tetstu et al., (PMID: 18498355)
8. Actin Antibody, Sigma, A2103. This antibody has been used and validated in our previous publication. (PMID: 27853189)
9. VE-Cadherin Antibidy, Cayman Chemicals, 160840: This antibody has been used in several publications. A recent publication's PMID is 31637214
10. VEGFR1 Antibody, Cell Signaling and Technology, 2479: This antibody has been used in several publications. A recent publication's PMID is 35701366

Eukaryotic cell lines

Policy information about cell lines and Sex and Gender in Research

Cell line source(s)

Human Umbilical Vein Endothelial cells (HUVECs) were purchased from Cell Applications. Primary Skeletal muscle microvascular endothelial cells and Bone marrow derived macrophages were isolated from male mice.

Authentication

Endothelial cells are authenticated by the expression of CD31 and VEGFR2. BMDMs were authenticated by their attachment to cell culture dishes, morphology and expression of F4/80

Mycoplasma contamination

Cell culture media was filtered through 0.1 micron filters to avoid mycoplasma contamination. Cells are free of Mycoplasma contamination

Commonly misidentified lines
(See ICLAC register)

Name any commonly misidentified cell lines used in the study and provide a rationale for their use.

Animals and other research organisms

Policy information about studies involving animals; ARRIVE guidelines recommended for reporting animal research, and Sex and Gender in Research

Laboratory animals

C57BL/6J, Balb/cJ male mice, 12-16 weeks old were used

Wild animals

Provide details on animals observed in or captured in the field; report species and age where possible. Describe how animals were caught and transported and what happened to captive animals after the study (if killed, explain why and describe method; if released, say where and when) OR state that the study did not involve wild animals.

Reporting on sex

We have considered sex when designing the study and chose male mice, as a previous study by Peng et al., (PMID: 21398592) have shown that male C57BL/6J mice show a robust angiogenic response to hind limb ischemia. Since our study used only male mice, the findings are applicable only to males. A total of 52 male mice were used for experiments that included laser scanning blood flow recovery, VEGF165b antibody administration, primary skeletal muscle microvascular endothelial cell isolation and developing bone marrow derived macrophages

Field-collected samples

For laboratory work with field-collected samples, describe all relevant parameters such as housing, maintenance, temperature, photoperiod and end-of-experiment protocol OR state that the study did not involve samples collected from the field.

Ethics oversight

University of Virginia and Augusta University Institutional Animal Ethical committee approved the protocol for the use of laboratory animals. Animal experiments were strictly adhered to NIH Animal use and care guidelines.

Note that full information on the approval of the study protocol must also be provided in the manuscript.
